# Does evidence support the high expectations placed in precision medicine? A bibliographic review

**DOI:** 10.12688/f1000research.13490.5

**Published:** 2019-06-10

**Authors:** Jordi Cortés, José Antonio González, María Nuncia Medina, Markus Vogler, Marta Vilaró, Matt Elmore, Stephen John Senn, Michael Campbell, Erik Cobo

**Affiliations:** 1 Department of Statistics and Operations Research, Universitat Politècnica de Catalunya, Barcelona, 08034, Spain; 2 Escuela Colombiana de Ingeniería Julio Garavito, Bogotá, 111211, Colombia; 3 Department of Statistics, Ludwig-Maximilians-Universität München, München, 80539, Germany; 4 Fundació lliga per a la investigació i prevenció del càncer, Reus, 43201, Spain; 5 Competence Center for Methodology and Statistics, Luxembourg Institute of Health, Strassen, 1445, Luxembourg; 6 School of Health and Related Research, University of Sheffield, Sheffield, S1 4DA, UK

**Keywords:** Constant Effect, Precision medicine, Homoscedasticity, Clinical Trial, Variability, Standard deviation, Review

## Abstract

**Background**: Precision medicine is the Holy Grail of interventions that are tailored to a patient’s individual characteristics. However, conventional clinical trials are designed to find differences in averages, and interpreting these differences depends on untestable assumptions. Although only an ideal, a constant effect of treatment would facilitate individual management. A direct consequence of a constant effect is that the variance of the outcome measure would be the same in the treated and control arms. We reviewed the literature to explore the similarity of these variances as a foundation for examining whether and how often precision medicine is definitively required.

**Methods**: We reviewed parallel clinical trials with numerical primary endpoints published in 2004, 2007, 2010 and 2013. We collected the baseline and final standard deviations of the main outcome measure. We assessed homoscedasticity by comparing the variance of the primary endpoint between arms through the outcome variance ratio (treated to control group).

**Results**: The review provided 208 articles with enough information to conduct the analysis. One out of five studies (n = 40, 19.2%) had statistically different variances between groups, implying a non-constant-effect. The adjusted point estimate of the mean outcome variance ratio (treated to control group) is 0.89 (95% CI 0.81 to 0.97).

**Conclusions**: The mean variance ratio is significantly lower than 1 and the lower variance was found more often in the intervention group than in the control group, suggesting it is more usual for treated patients to be stable. This observed reduction in variance might also imply that there could be a subgroup of less ill patients who derive no benefit from treatment. This would require further study as to whether the treatment effect outweighs the side effects as well as the economic costs. We have shown that there are ways to analyze the apparently unobservable constant effect.

## Introduction

The goal of precision medicine is to develop prevention and treatment strategies that take into account individual characteristics. As Collins and Varmus stated, “The prospect of applying this concept broadly has been dramatically improved by recent developments in large-scale biologic databases (such as the human genome sequence), powerful methods for characterizing patients (such as proteomics, metabolomics, genomics, diverse cellular assays, and mobile health technology), and computational tools for analyzing large sets of data.” With this words in mind, US President Obama gave his strong endorsement in launching the 2015 Precision Medicine initiative to capitalize on these developments ^1, 2^. Here, we aim to quantify the proportion of interventions that may benefit from this idea.

**Figure 1.**
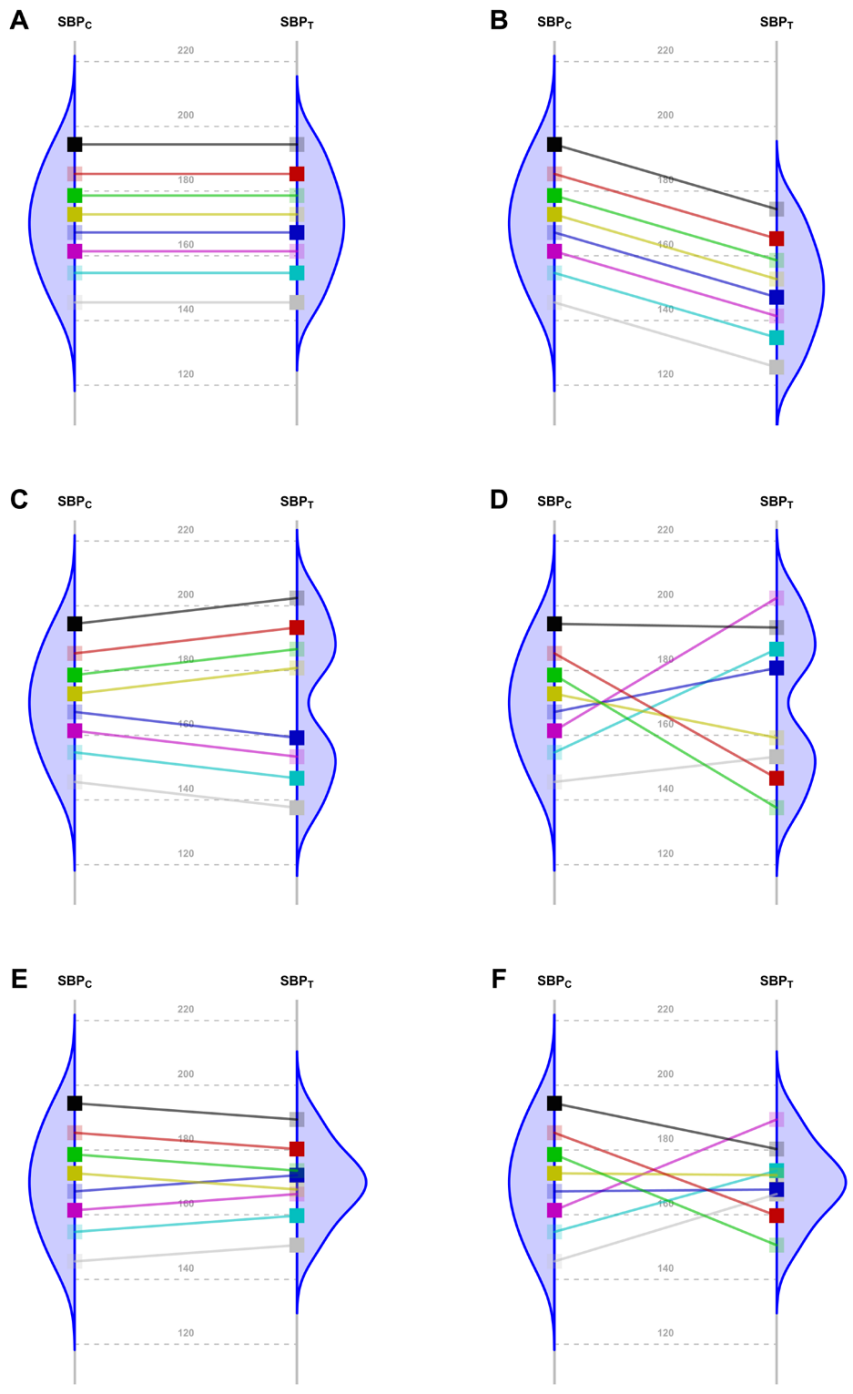
Scenarios representing fictional trials using 8 participants with systolic blood pressure as the primary endpoint. Because of the random allocation to one of two treatment arms, we will observe only one of the two potential outcomes for each patient: either under T or under C. Fully saturated colors represent observed systolic blood pressure (SBP) values, and transparent squares represent missing potential SBP values. The line slope indicates the individual non-observable effect for each patient. Densities are the potential distributions of the outcome in each group: As both random samples come from the same target population, the average causal effect is estimable without bias. **Panel A** shows the potential outcome values that we could obtain if there were not any treatment effect; as the intervention has no effect at all, both groups have the same distribution (i.e., mean and variance). **Panel B** shows the scenario of a constant effect, meaning that the intervention lowers the SBP by a single value in every patient and thus implying the same variability in both arms. For instance, the study from Duran-Cantolla *et al.* ^3^ compared the 24-hour SBP in 340 patients randomized to either continuous positive airway pressure (CPAP) or sham–CPAP, and they observed a greater decrease of 2.1 mmHg (95% CI from 0.4 to 3.7) in the intervention group compared to the control group. Furthermore, baseline standard deviations (SDs) were 12 and 11; and final SDs were 13 for both groups. Therefore, their results fully agree with the trial design’s assumption of a constant effect (scenario B) and nothing contradicts the inference that each patient exhibits a constant reduction of 2.1mmHg, although uncertainty from sampling makes the results compatible with a constant effect that lies somewhere between 0.4 and 3.7. **Panel C** represents a situation with 2 different effects in 2 subpopulations (“treatment by subgroup interaction”). Although the effects are identical within them, the observable distribution in the treated arm would have higher variability. Here, finer eligibility criteria for classifying patients in those subpopulations might allow us to assume a constant effect again. In **Panel D**, the treatment has a variable effect in each patient, resulting also in greater variability within the treated arm but without any subgroup sharing a common effect. The results are poorly predictive about the effects on future patients. In the study by Kojima *et al.* ^4^, the primary outcome measure was the 3-hour postprandial area under the curve of apolipoprotein B48, with outcome SDs being, respectively, 0.78 and 0.16 in the treated and reference arms, thus showing an outcome variance ratio of 23.77. This is compatible with different treatment effects that could need additional refinements through precision medicine, since a greater variance in the treated arm indicates that “ *the interpretation of the main treatment effect is controversial”* ^5^. In that case, guidelines for treating new patients should be based either on additional eligibility criteria (“precision medicine”, panel C) or on n-of-1 trials (“individualized medicine”, panel D) ^6– 10^. W. S. Gosset already highlighted this “treatment by patient interaction” in his 1908 paper, where he introduced the Student t-distribution ^11^. Alternatively, interactions can result in smaller variances in the treated arm. **Panel E** shows a different effect in 2 subgroups; but the variability is now reduced, thus indicating that the best solution would be to identify the subpopulations in order to refine the selection criteria. In **Panel F**, the treatment again has a variable effect on each patient; but unlike Panel D, in this case the consequence is less variability within the treated arm. In the study from Kim *et al.* ^12^, the primary endpoint was the PTSD Checklist–Civilian Version (PCL-C). This scale is based on the sum of 17 Likert-scale symptoms, ranging from 17 (perfect health) to 85 (worst clinical situation). At the end of the trial, the respective outcome SDs were 16 and 3 for the control and treated arms, meaning that variance was reduced around 28 times. This situation can correspond to scenarios E or F, and it merits statistical consideration, that is beyond the scope of this paper.

The fundamental problem of causal inference is that for each patient in a parallel group trial, we can know the outcome for only one of the interventions. That is, we observe their responses either to the new treatment or to the control, but not both. By experimentally controlling unknown confounders through randomization, a clinical trial may estimate the averaged causal effect. In order to translate this population estimate into effects for individual patients, additional assumptions are needed. The simplest and strongest one is that the effect is constant. Panels A and B in Figure 1 ^3– 12^ represent two scenarios with a common effect in all patients, although it is null in the first case. Following Holland ^13^, this assumption has the advantage of making the average causal effect relevant to each patient. All other scenarios ( Figure 1, Panels C to F) require additional parameters to fully specify the treatment effect.

As an example, the 10 clinical trials published by the journal Trials in October 2017 ( Supplementary File 1: Table S1) were designed without explicitly allowing for an effect that was not constant within the study population. Furthermore, all their analyses intended to estimate just an average effect with no indication of any possible interaction with baseline variables ( Figure 1, Panels C and E), nor did they discuss any random variability for the treatment effect ( Figure 1, Panels D and F). Therefore, without further specifications, it seems that they were either hoping for the treatment effect to be the same for all patients or assuming that it was not useful to try and investigate this. As a contrary example, Kim *et al*. ^14^ designed their trial to test an intervention for: 1) non-inferiority in the overall population and 2) superiority in the subgroup of patients with high epidermal growth factor receptor expression.

The variability of a clinical trial outcome measure is relevant because it conveys important information about whether or not precision medicine is achievable. Does variance come only from unpredictable sources of patient variability? Or should it also be attributed to different treatment effects that require more precise prescription rules ^15– 17^? One observable consequence of a constant effect is that the treatment will not affect variability, and therefore the outcome variances in both arms should be equal (“homoscedasticity”).

Below, we will elucidate whether the comparison of observed variances may shed some light on the non-observable individual treatment effect.

Our objectives are, first, to compare the variability of the main outcome between arms in parallel randomized controlled trials published in medical journals; and, second, to provide a rough estimate of the proportion of studies that could potentially benefit from precision medicine. To assess the consistency of results, we also explore the evolution of the variability of the treated arm over time (from baseline to the end of the study).

## Methods

### Population

Our target population was parallel, randomized controlled trials with numerical primary endpoint. The trials should provide enough information to assess two homoscedasticity assumptions in the primary endpoint: between arms at trial end; and baseline to outcome over time in the treated arm. Therefore, baseline and final SDs for the main outcome were necessary or, lacking those, we required at least one measure that would allow us to calculate them (variances, standard errors or mean confidence intervals).

### Data collection

Using the Medline database, we selected articles on parallel clinical trials from the years 2004, 2007, 2010 and 2013 with the following criteria: “ AB (clinical trial* AND random*) AND AB (change OR evolution OR (difference AND baseline))” [The word “difference” was paired with “baseline” because the initial purpose of the data collection (although it was subsequently modified) was to estimate the correlation between baseline and final measurements]. The rationale behind choosing these years was to have a global view of the behavior of the studies over a whole decade. For the years 2004 and 2007, we selected all papers that met the inclusion criteria. However, we retrieved a greater number of articles from our search for the years 2010 and 2013 (478 and 653, respectively); therefore, we chose a random sample of 300 papers (Section II in Supplementary File 1).

Data were collected by two researchers (NM, MkV) in two phases: 2004/2007 and 2010/2013. Later, two statisticians (JC, MtV) verified the data and made them accessible to readers through a *Shiny* application and through the *Figshare* repository ^18^.

### Variables

Collected variables were: baseline and outcome SDs; experimental and control interventions; sample size in each group; medical field according to *Web of Science* (WOS) classification; main endpoint; indication; type of disease (chronic versus acute); endpoint type (measured versus scored); intervention type (pharmacological versus non-pharmacological); improvement direction (positive versus negative); and whether or not the main effect was statistically significant.

For studies that reported more than one numerical endpoint and failed to clarify which endpoint was the primary endpoint, the latter was determined using the following hierarchical criteria: (1) objective or hypothesis; (2) sample size determination; (3) main statistical method; (4) first numerical variable reported in results.

In the same way, the choice of the "experimental" arm was determined depending on its role in the following sections of the article: (1) objective or hypothesis; (2) sample size determination; (3) rationale in the introduction; (4) first comparison reported in results (in the case of more than two arms).

### Statistical analysis

We assessed homoscedasticity between treatments and over time. For the former, our main analysis compared the outcome variability between treated (T) and control (C) arms at the end of the trial. For the latter, we compared the variability between outcome (O) and its baseline (B) value for the treated arm.

Three different methods were used to compare the variances: 1) a random-effects model; 2) a heuristic procedure based on the heterogeneity obtained from the previous random-effects model; and 3) a classical test for equality of variances.

To distinguish between the random sampling variability and heterogeneity, we fitted a random-effects model. The response was the logarithm of the outcome variance ratio at the end of the trial. The covariates were the study as a random effect, while the logarithm of the variance ratio at baseline served as a fixed effect ^19^.

The main fitted model for between-arm comparison was:

$$\log{\left(\frac{V_{OT}}{V_{OC}}\right)}_{i}=\mu +S_{i}+\beta .\log{\left(\frac{V_{BT}}{V_{BC}}\right)}_{i}+e_{i}$$

$$with\;S_{i}∼N\left(0,\;\tau^{2}\right)\;and\;e_{i}∼N\left(0,\;v_{i}^{2}\right)$$

where V ij represents the variances of the outcome in each arm (V _iT,_ V _iC_) at the end of the study (V _OT,_ V _OC_) and at baseline (V _BT,_ V _BC_). The parameter *μ* is the logarithm of the average variance ratio across all the studies; *s*_i_ represents the heterogeneity of the between-study effect associated with study *i* and having variance τ ^2^; β is the coefficient for the linear association with the baseline variance ratio; and e _i_ represents the intra-study random errors with variance vi2 .

The parameter *μ* represents a measure of the imbalance between the variances at the end of the study, which we call heteroscedasticity.

The estimated value of τ ^2^ provides a measure of heterogeneity, that is, to what extent the value of *μ* is applicable to all studies. The larger τ ^2^ is, the lesser the homogeneity.

The percentage of the response variance explained by the differences among studies in respect to the overall variance is measured by the *I* ^2^ statistic ^20^. That is:

$$I^{2}=\frac{\tau^{2}}{\tau^{2}+v^{2}}$$

*v* ^2^ is the mean of the error variances $v_{i}^{2}$.

An analogous model was employed to assess the homoscedasticity over time. As there is only one available measure for each study, it is not possible to differentiate both sources of variability: (i) within-study or random variability; and (ii) heterogeneity. To isolate the second, the first was theoretically estimated using either the delta method, in the case of comparison between arms, or some approximation, in the case of comparison over time (see details in Sections VI and VII of Supplementary File 1). Thus, the within-study variance was estimated using the following formulas:

$$V\left[log\;\left(\frac{V_{OT}}{V_{OC}}\right)\right]=\frac{2}{n_{OT}-2}+\frac{2}{n_{OC}-2}\;\;\;(between\;\;arms)$$

$$V\left[log\;\left(\frac{V_{OT}}{V_{BT}}\right)\right]=\frac{4}{n-1}-2\cdot log\;\left[\text{1}+\frac{2\cdot Corr{\left[Y_{OT},Y_{BT}\right]}^{2}}{n^{2}/(n-1)}\right]\;\;\;(over\;\;time)$$

Funnel plots centered at zero are reported in order to help investigate asymmetries. They represent the variance ratios as a function of their standard errors. The first and main analysis considers the studies outside the triangle delimited by ± 2 times the standard error to be those that have statistically significant differences between variances.

The second analysis is heuristic. In order to obtain a reference value for τ ^2^ in the absence of treatment effect, we first modeled the baseline variance ratio as a response that is expected to have heterogeneity equal to 0 due to randomization – provided no methodological impurities are present (e.g., considering the outcomes obtained 1 month after the start of treatment to be the baseline values). This *reference* model allows us to know the proportion of studies in the previous models that could increase heterogeneity over levels that are incompatible with a constant effect situation. (Section III in Supplementary File 1). Specifically, studies with larger discrepancies in variances were removed one by one until the estimated value of τ was as close as possible to that of the *reference* model. These deleted studies were considered to be those that had significantly different variances, perhaps because the experimental treatment either increased or decreased the variance. From now on, the complete dataset and the resulting dataset after removing the abovementioned studies will be called CDB (complete dataset) and RDB (reduced dataset) for between-arm comparison and CDO (Complete) and RDO (Reduced) for over-time comparison.

Thirdly, as an additional sensitivity analysis, we also assessed homoscedasticity in each single study by using tests for comparing variances: (a) between outcomes in both arms with an F-test for independent samples; and (b) between baseline and outcome in the treated arm with a test for paired samples ^21^ when the variance of the paired difference was available. All tests were two-sided (α=5%).

Several subgroup analyses were carried out according to the statistical significance of the main treatment effect and to the different types of outcomes and interventions.

All analyses were performed with the R statistical package version 3.2.5. (The R code for the main analysis is available from https://doi.org/10.5281/zenodo.1239539 ^22^)

## Results

### Population

**Figure 2.**
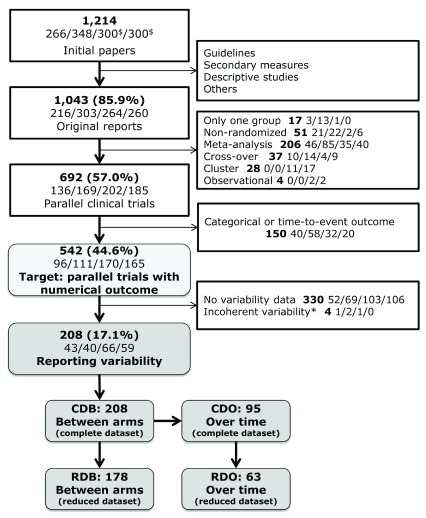
Flow-chart of the articles in the study. Percentages represent the number of papers with respect to the ones retrieved from the bibliographic search. The number of articles for each year (2004/2007/2010/2013) is specified in the second line of each box (separated by slashes). ^$^300 papers were randomly selected for years 2010 and 2013. *Four papers were excluded because the variance of the change over time was inconsistent with both the baseline and final variances, which would lead to impossible absolute correlation estimates greater than 1. CDB and RDB are the datasets used in the main and heuristic analysis, respectively, for the between-arm comparison. CDO and RDO are the datasets used in the main and heuristic analysis, respectively, for the over-time comparison.

A total of 1214 articles were retrieved from the search. Of those papers, 542 (44.6%) belong to the target population and 208 (17.1%) contained enough information to enable us to conduct the analysis ( Figure 2).

The majority of the selected studies were non-pharmacological (122, 58.6%); referred to chronic conditions (101, 57.4%); had a continuous outcome measured with units (132, 63.8%) instead of a constructed scale; had an outcome that was measured (125, 60.1%) rather than assessed; and had lower values of the outcome indicating positive evolution (141, 67.8%). Regarding the primary objective of each trial, the authors found statistically significant differences between arms (all of which favored the treated group) in 83 (39.9%) studies. Following the Web of Science criteria, 203 articles (97.6%) belonged to at least one medical field. The main areas of study were: General & Internal Medicine (n=31, 14.9%), Nutrition & Dietetics (21, 10.1%), Endocrinology & Metabolism (19, 9.1%), and Cardiovascular System & Cardiology (16, 7.7%).

### Homoscedasticity

In descriptive terms, the average of the outcome variance ratio is 0.94, reflecting lower variability in the treated arm. At the end of the study, 113/208 (54%, 95% CI, 47 to 61%) papers showed less variability in the treated arms ( Supplementary File 1: Figure S1 and Figure S2). Among the treated arms, 111/208 (53%, 95% CI, 46 to 60%) had less or equal variability at the end of follow-up than at the beginning ( Supplementary File 1 : Figure S3 and Figure S4).

**Figure 3.**
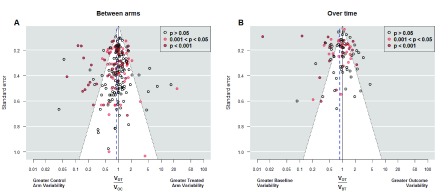
Funnel plots of variance ratio. Funnel plots of outcome variance ratio between arms ( **Panel A**) and of outcome variance ratio over time ( **Panel B**). The first shows all 208 studies while the second shows only the 95 studies in which the variance of the difference between the baseline and final response was available. Vertical axis indicates precision for the comparison of variances; with points outside the triangle being statistically significant. Additionally, red points mark significant differences between the means, which correspond to each study’s objective to assess main treatment effects. In **Panel A**, points on the right indicate higher outcome variability for the treated individuals, as expected if there is patient-by-treatment interaction; similarly, points on the left correspond to lower variability, although this is compatible with traditional Evidence-Based Medicine. Eleven (5.2%) out of 208 studies reported exactly the same outcome variability in both arms. We observe more red points on the left, indicating that changes in the average accompany reductions in the variance. In **Panel B**, points on the right indicate higher variability in the treated arm at the end of the study, as expected in a scenario of heterogeneous treatment effect; points on the left correspond to lower variability at the end, which implies a more homogenous response after treatment. The largest number of points on the left side indicates a majority of experimental interventions that reduce variability. In addition, several of these interventions yielded significant results in the main endpoint. V _OT_: variance of the outcome in the treated arm. V _OC_: variance of the outcome in the control arm. V _BT_: variance of the outcome at baseline in the treated arm.

Based on the random-effects model ( Supplementary File 1: Table S4, model 3 with CDB) the adjusted point estimate of the mean outcome variance ratio for comparison between arms (Treated to Control group) is 0.89 (95% CI 0.81 to 0.97). This indicates that treatments tend to reduce the variability of the patient's response by about 11% on average. As for the comparison over time ( Supplementary File 1 : Table S4, Model 6 with CDO), the average variability at the end of the studies is 14% lower than that at the beginning. Figure 3 shows the funnel plots derived from the random-effects models. The triangles delimit the 95% confidence regions of random variability. In the between-arm comparison, the studies (represented by the circles) to the right of the triangle have variances that are significantly larger in the treatment arm than in the control arm, while those on the left are significantly larger in the control arm. As for the over-time comparison, the studies to the right have a significantly higher variance at the end of the study in the treated group, while those on the left are significantly larger at the beginning of the study. Table 1 ( *random-effects* method) shows the frequencies and percentages of the studies according to the classification illustrated in these funnel plots.

**Table 1.** Variance comparison. Alternative possible methods for estimating the number and percentage of studies with different variances on comparisons between arms and over-time. Limits for declaring different variances come from different statistical methods: (1) the analysis relying on random-effects model and funnel plots; (2) the heuristic analysis based on number of studies that have to be deleted from the random-effects model in order to achieve a negligible heterogeneity (studies with larger discrepancies in variances were removed one by one until the estimated value of τ was as close as possible to that of the reference model – the one that compares the variances of the response at baseline. See Methods for details); (3) classic statistical tests for comparing variances (F for independent outcomes or Sachs’ test ^21^ for related samples). ^¥^ This comparison was performed on studies reporting enough information to obtain the variability of the change from baseline to outcome, for example because they provide the correlation between outcome and baseline values.

Comparing variances	N	Method	After treatment, variability is…		
			Increased n (%)	Decreased n (%)	Not changed n (%)
Outcome between treatment arms	208	Random-effects model	14(6.7%)	26 (12.5%)	168(80.8%)
		Heuristic	11 (5.3%)	19 (9.1%)	178 (85.6%)
		F-test	15 (7.2%)	26 (12.5%)	167 (80.3%)
Outcome versus baseline in treated arm	95 ^¥^	Random-effects model	16 (16.8%)	22(23.2%)	57(60.0%)
		Heuristic	13 (13.7%)	19 (20.0%)	63 (66.3%)
		Paired test	16 (16.8%)	22 (23.2%)	57 (60.0%)

The second heuristic analysis was motivated by the fact that the estimated baseline heterogeneity (τ ^2^) was 0.31 ( Supplementary File 1 : Table S4, Model 1 with CDB), which is a very high value that could be explained by methodological flaws similar to those presented by Carlisle ^23^. Fortunately, the exclusion of the four most extreme papers reduced it to 0.07 ( Supplementary File 1 : Table S4, Model 1 with RDB); one of these was the study by Hsieh *et al.* ^24^, whose “baseline” values were obtained 1 month after the treatment started. When we modeled the outcome instead of the baseline variances as the response, estimated heterogeneity (τ^=0.55) was almost doubled ( Supplementary File 1 : Table S4, Model 6 with CDB). We found 30 studies that compromised homoscedasticity: 11 (5.3%) with higher variance in the treated arm and 19 (9.1%), with lower variance (see *heuristic* method in Table 1). Based on the classical variance comparison tests (sensitivity analysis), these figures were slightly higher: 41 studies (19.7%) had statistically significant differences between outcome variances; 15 (7.2%) favored greater variance in the treated arm; and 26 (12.5%) were in the opposite direction. Larger proportions were obtained from the comparisons over time of 95 treated arms: 16.8% had significantly greater variability at the end of the study and 23.2% at the beginning. Table 1 also summarizes those numbers for the *F-test* and *paired Test*.

Subgroup analyses suggest that significant interventions had an effect on reducing variability ( Supplementary File 1 : Figures S5–S7), a fact which has already been observed in other studies ^25, 26^. Even more importantly, lower variances in the treated arm occur only in outcomes for which a positive response is defined as a decrease from baseline. This is in line with other works that have found a positive correlation between the effect size and its heteroscedasticity ^27, 28^. The fact is that it is difficult to find heteroscedasticity when there is no overall treatment effect. The remaining subgroup analyses did not raise concerns (Section V in Supplementary File 1).

## Discussion

### Main findings

We aimed to show that comparing variances provides evidence about whether or not precision medicine is a sensible choice. When both arms have equal variances, then a simple and believable interpretation is that the treatment effect is constant, which, if correct, would render futile any search for predictors of differential response. This means that the average treatment effect can be seen as an individual treatment effect (not directly observable), which supports the use of a unique clinical guideline for all patients within the eligibility criteria, thus in turn also supporting the use of parallel controlled trials to guide decision-making in these circumstances. Otherwise, heteroscedasticity may suggest a need to specify further the eligibility criteria or search for an additive scale ^25, 29^. Because interaction analyses cannot include unknown variables, there might be value in repeating trials once any new potential interaction variable emerges (e.g., a new biomarker) as a candidate for a new subgroup analysis. We have described how homoscedasticity can be assessed when reporting trials with numerical outcomes, regardless of whether every potential effect modifier is known.

We have provided a rough estimate of the proportion of interventions with different variability that might benefit from more precise medicine: Considering the most extreme result from Table 1 for comparison between arms, 1 out of 14 interventions (7.2%) had greater variance in the treated arm while 1 out of 8 interventions (12.5%) had lower variance. That is, we have found evidence of effect variation in only 1 out of 5 trials (40/208), suggesting a limited role for tailored interventions. These might be pursued by either a finer selection criteria (common effect within specific subgroups), or with n-of-1 trials (no subgroups of patients with a common effect).

The sensitivity analysis of the change over time in the treated arm agreed with the findings in the comparison between arms, although this comparison is not protected by randomization. For example, the existence of eligibility criteria at baseline may have limited the initial variance (a hypertension trial might recruit patients with baseline SBP between 140 and 159 mm Hg), leading to the variance increasing naturally over time.

Regarding the subgroup analyses, we found that variability seems to decrease for treatments that perform significantly better than the reference; otherwise, it remains similar. Therefore, the treatment seems to be doing what medicine should do: having larger effects in the most ill patients. Two considerations may be highlighted here: (1) as the outcome range becomes reduced, we may interpret that, following the intervention, this population is under additional control; but also, (2) as subjects are responding differently to treatment, this opens the way for not treating some (e.g., those subjects who are not very ill and thus lack the scope to respond very much), which subsequently incurs savings in side effects and costs.

This reduced variability could also be due to methodological reasons. One is that some measurements may have a “ceiling” or “floor” effect (e.g., in the extreme case, if a treatment heals someone, no further improvement is possible). In fact, according to the subgroup analysis of the studies with outcomes that indicate the degree of disease (high values imply greater severity; e.g., pain), a greater variance (25%) is obtained in the treated arm (see Figure S5). However, in the studies with outcomes that measure the degree of healthiness (high values imply better condition; e.g., mobility), the average variances match between arms, and this does not suggest a ceiling effect. As mentioned above, another reason might be that the treatment effect is not additive on the scale used for analysis, suggesting that it would be suitable to explore other metrics and transformations. For example, if the treatment acts proportionally rather than linearly, the logarithm of the outcome would be a better scale.

## Limitations

There are three reasons why these findings do not invalidate precision medicine in all settings. First, there are studies where the variability in the response is glaringly different, indicating the presence of a non-constant effect. Second, the outcomes of some type of interventions such as surgeries, for example, are greatly influenced by the skills and training of those administering the intervention; and these situations could have some effect on increasing variability. And, third, this study focuses on numerical endpoints; thus, time-to-event or categorical outcomes are out of scope.

The results rely on published articles, which raises some relevant issues. First, some of our analyses are based on Normality assumptions that are unverifiable without access to raw data. Second, a high number of manuscripts (61.6%, Figure 2) act contrary to CONSORT ^30^ advice in that they do not report variability. Thus, the included studies may not be representative. Third, trials are usually powered to test constant effects and thus the presence of greater variability would lead to an underpowered design; that is, if the control group variance is used to plan the trial, increased treatment group variance would reduce power (perhaps leading to non-publication). Fourth, the heterogeneity observed in the random-effects model may be the result of methodological inaccuracies ^23^ arising from typographical errors in data translation, inadequate follow-up, insufficient reporting, or even data fabrication. On the other hand, this heterogeneity could also be the result of relevant undetected factors interacting with the treatment, which would indeed justify the suitability of precision medicine. A fifth limitation is that many clinical trials are not completely randomized. For example, multicenter trials often use a permuted blocks method. This means that if variances are calculated as if the trial were completely randomized (which is standard practice), the standard simple theory covering the random variation of variances from arm to arm is at best approximately true ^25^

**Figure 4.**
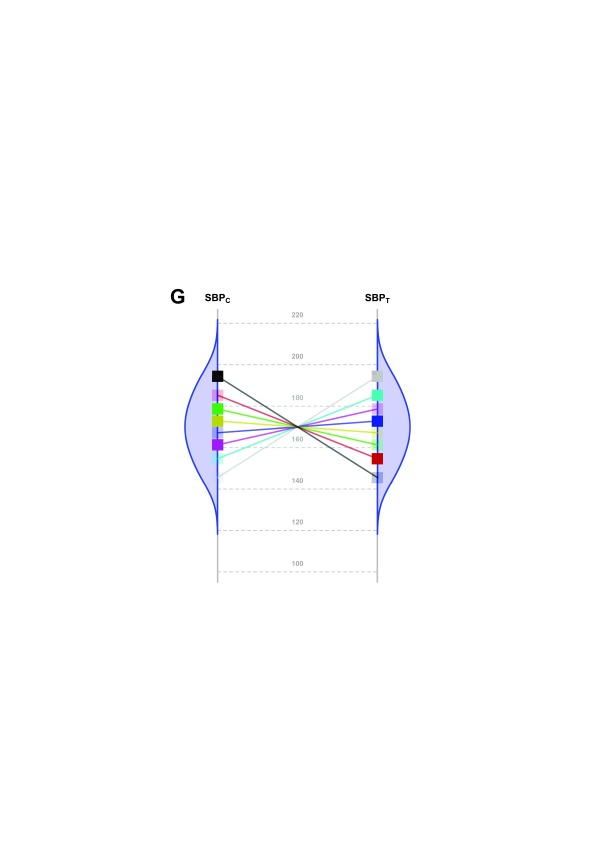
Scenario representing a fictional trial with 8 participants and having homoscedasticity but a non-constant effect. SBP potential values of each patient in both groups (C: control; T: treated) under a highly hypothetical scenario: the treatment effect has no value if systematically applied to the whole population; but if n-of-1 trials could be performed in this situation, the best treatment strategy would be chosen for each patient and the overall health of the population would be improved.

The main limitation of our study arises from the fact that, although a constant effect always implies homoscedasticity on the chosen scale, the reverse is not true; i.e., homoscedasticity does not necessarily imply a constant effect. For example, the highly specific and non-parsimonious situation reflected in Figure 4 indicates homoscedasticity but without a constant effect. Nevertheless, a constant effect is the simplest explanation for homoscedasticity (Section VIII of Supplementary File 1: Conditions for homoscedasticity to hold without a constant effect under an additive model).

## Conclusion

In summary, for most trials, the variability of the response to treatment scarcely changes or even decreases. Thus, if we take into account the limitation previously explained in Figure 4, this suggests that the scope of precision medicine may be less than what is commonly assumed. Evidence-Based Medicine (EBM) operates under the paradigm of a constant effect assumption, by which we learn from previous patients in order to develop practical clinical guidelines for future treatments. Here, we have provided empirical insights to postulate that such a premise is reasonable in most published parallel randomized controlled trials. However, even where one common effect applies to all patients fulfilling the eligibility criteria, this does not imply that the same decision is optimal for all patients. More specifically, this is because different patients and stakeholders may vary in their weighting not only of efficacy outcomes, but also of the harm and cost of the interventions – thus bridging the gap between common evidence and personalized decisions.

Our results uphold the assertion by Horwitz *et al.* that there is a “need to measure a greater range of features to determine [...] the response to treatment” ^31^. One of these features is an old friend of statisticians, the variance. Looking only at averages can cause us to miss out on important information.

## Data availability

Data is available through two sources:

- A shiny app that allows the user to interact with the data without downloading it: http://shiny-eio.upc.edu/pubs/F1000_precision_medicine/
- The Figshare repository: https://doi.org/10.6084/m9.figshare.5552656 ^18^

In both sources, the data can be downloaded under a Creative Commons License v. 4.0.

The code for the main analysis is available at the following link: https://doi.org/10.5281/zenodo.1239539 ^22^

## Supplementary material

**Supplementary File 1: The supplementary material contains the following sections**

- Section I: Constant effect assumption in sample size rationale

- Section II: Bibliographic review

- Section III: Descriptive measures

- Section IV: Random-effects models

- Section V: Subgroup analyses

- Section VI: Standard error of log(V _OT_/V _OC_) in independent samples

- Section VII: Standard error of log(V _OT_/V _BT_) in paired samples

- Section VIII: Conditions for homoscedasticity to hold without a constant effect under an additive model
