# Supplementary material for: Does evidence support the high expectations placed in precision medicine? A bibliographic review
Source: F1000Res. 2019 Jun 10;7:30. Originally published 2018 Jan 9. [Version 5] doi: 10.12688/f1000research.13490.5 (PMC6524747; doi:10.12688/f1000research.13490.5)
Supplement: Supplementary file 1 [file f1000research-7-21429-s0000.tgz › 9a9383bb-90d4-4de1-83ab-edee1fd9850a_Supplementary_File_1_v5.pdf]

Supplementary appendix

Table of Contents

Section I: Constant effect assumption in sample size rationale....................................... 2

Section II: Bibliographic review.. 6

Section III: Descriptive measures 8

Section IV: Random-effects models 11

Section V: Subgroup analyses 14

Section VI: Standard error of $log(V_{OT}/V_{OC})$ in independent samples 18

Section VII: Standard error of $log(V_{OT}/V_{BT})$ in paired samples 21

Section VIII: Conditions for homoscedasticity to hold without a constant effect under an additive model 26

References 28

Section I: Constant effect assumption in sample size rationale

Researchers, care providers and policy makers probably do not realize that behind the classical approach of evidence-based medicine there is the assumption of a constant effect within a targeted population. For example, we have reviewed the last 10 protocols of clinical trials published in Trials Journal (1-10) in October 2017: From the 10 selected protocols, 2 were pilot studies with objectives more related to trial logistic feasibility. From the 8 protocol devised to test intervention efficacy, 8 out of 8, assumed an unique, fixed or constant effect (Table S1). In other words, the common premise behind their sample size rationale is that there is no need to further refine the application of the intervention so long as the patient fulfils the eligibility criteria.

Under this design assumption, evidence-based-medicine decisions have led to great progress and allowed for social agreements such as the provision of economic revenue for developers of new medical interventions, whether they be drugs or devices. However, when the intervention requires a different effect for each individual, questions arise on how to agree on a price (e.g., whether it should be either fixed or variable) prior to market authorization.

Table S1.The last ten randomized clinical trials protocols published in October 2017 in the Trials Journal. The last column (*Sample size*) includes the paragraph of the text of the sample size calculation in which a constant treatment effect is assumed. Specific sentences are highlighted in bold.

Date	Title	Sample size	Statistical analysis
Oct 23	A multi-centre randomised trial to compare the effectiveness of geriatrician-led admission avoidance hospital at home versus inpatient admission	Our proposed study effect estimate is based on a control group (inpatient admission) event rate at 12 months of 50% living in a residential setting, **with a 10% reduction** to 40% in the admission avoidance hospital at home group, equal to a relative risk of 0.8	In the model, participants and centres will be fitted as random effect, and time **and treatment as fixed effect**. We will fit an interaction term between time and intervention group so that possible differences of intervention effect could be assessed at each time point
Oct 25	Neoadjuvant everolimus plus letrozole versus fluorouracil, epirubicin and cyclophosphamide for ER-positive, HER2-negative breast cancer: study protocol for a randomized pilot trial	Because of the exploratory (pilot) nature of this study, statistical power was not calculated to assess specific study outcomes […]. **This sample size calculation was developed in accordance with the recommendations by previous reports on sample size determination** for pilot studies	The ultrasound response rate, pCR rate, breast-conserving surgery rate, and toxicities will be compared between the two arms using the chi-square test.
Oct 27	Long-term Effects of high-doSe pitavaStatin on Diabetogenicity in comparison with atorvastatin in patients with Metabolic syndrome (LESS-DM): study protocol for a randomized controlled trial	The sample size was calculated by **assuming an expected difference of 0.2%** in hemoglobin A1c change between the groups and a population variance of 0.5%, based on previous studies.	The difference in changes between the groups will be analyzed using both Student’s t test and analysis of covariance to adjust for the baseline values

Oct 26	Safety of tubal ligation by minilaparotomy provided by clinical officers versus assistant medical officers: study protocol for a noninferiority randomized controlled trial in Tanzanian women	Assuming a 3% major AE rate in the control group (AMOs), we will demonstrate **noninferiority within the margin of 2%** at a one-sided significance level of α = 0.05 and a power of 80% (calculated when AE rates in both arms are the same) with a sample size of 895 per arm (1790 women in total).	Independent sample t test [Table 1]
Oct 27	Improving oxygen therapy for children and neonates in secondary hospitals in Nigeria: study protocol for a stepped-wedge cluster randomised trial	Based on an alpha of 5%, we calculated that we would have approximately 80% power **to detect a 35% reduction** in pneumonia case fatality rate (6.1% to 4%), and 90% power to detect a 20% reduction in neonatal case fatality rate (8.2% to 6.6%)	We will adjust for the effect of calendar time, clustering, and other significant confounders using generalised linear mixed models (GLMM) or generalised estimating equations (GEE) [32, 44]. We **will examine heterogeneity in effects between different hospitals** using within-cluster comparisons of exposed and unexposed periods...
Oct 30	SCORE: Shared care of Colorectal cancer survivors: protocol for a randomised controlled trial	Evidence of a substantial detriment associated with shared care as **measured by a reduction of 0.6** (or worse) on a patient-reported outcome measure would be detected with 80% power (at the 2.5% one-sided level of significance)	The effect of shared care compared with usual care on scales from the patient-reported outcome measures will be quantified by applying a mixed model for repeated measures approach to the data collected. Point estimates of effect on these scales will be presented with 95% CIs
Oct 23	Efficacy of inhaled HYdrogen on neurological outcome following Brain Ischemia During post-cardiac arrest care (HYBRID II trial): study protocol for a randomized controlled trial	On the basis of published data […] and **assumed that the absolute risk reduction by HI is 15%**; that is, the favourable neurological outcome rate improves from 50% to 65% with HI. A sample size of 167 patients in each group will provide 80% power to detect a 15% change in the proportion of good neurological outcomes (CPCs of 1 and 2), from 50% to 65%,	The proportion of patients achieving good neurological outcomes at the end of the follow-up will be compared using the Pearson $\chi 2$ test, which will be the primary result of the trial

Oct 27	The effects of exercise on the quality of life of patients with breast cancer (the UMBRELLA Fit study): study protocol for a randomized controlled trial	[…] we assume a 6-point increase in QoL in the control group and a 16-point increase in the intervention group, among patients who accept the intervention in this cmRCT. We expect an attendance rate of 70% in the intervention group and assume that the improvement in non-attenders randomized to the intervention group (30%) is equal to the improvement in the control group (i.e. 6 points). Furthermore, we assume that non-attendance does not impact the standard deviation. As a result, **we estimate a mean improvement of 13 points in the intervention group** ((70*16 + 30*6)/100 = 13) instead of 16 points **and a mean improvement of 6 points in the control group**.	To assess contrasts in physical activity levels between the intervention and control group, we will compare changes in physical activity levels within the control group to changes in the intervention group using analysis of covariance (ANCOVA) or mixed linear regression models adjusted for baseline for the analyses of repeated outcomes
Oct 24	A randomised controlled trial assessing the severity and duration of depressive symptoms associated with a clinically significant response to sertraline versus placebo, in people presenting to primary care with depression (PANDA trial): study protocol for a randomised controlled trial	The results from previous metaanalyses suggest that the effect size of SSRIs versus placebo is **about an 11% reduction in the Hamilton Rating Scale** for Depression (HAM-D) score […] Our best estimate of the minimal clinically important difference (MCID) from the PANDA cohort study is that this corresponds to a **14 percentage points (95% CI 10 to 17 percentage points) reduction in score on the PHQ-9** […] Therefore giving the power for effect sizes of 11 and 14 percentage points is reasonable and conservative in the light of the confidence limits and the previous results from the systematic review.	We will use a mixed-effects generalised linear modelling framework as it is appropriate for repeated measures data [38, 39]. The analyses will include adjustments for baseline PHQ-9 score and the stratification variables (severity in three categories, duration in two categories and centre)
Oct 30	Targeting low- or high-normal Carbon dioxide, Oxygen, and Mean arterial pressure After Cardiac Arrest and REsuscitation: study protocol for a randomized pilot trial	In a previous, small RCT, the use of 30% FiO2, compared with 100% FiO2, resulted in approximately 50% increase of NSE values at 48 h in the subset of patients treated with hypothermia. Assuming this previous finding, a study with 39 patients in each arm would have a power of 80%, with the significance set at 0.05, **to detect a 50% increase in NSE.**	Low-normal and high-normal PaCO2, PaO2, and MAP values will be compared over time using a generalized mixed model with a compound-symmetry covariance matrix

Section II: Bibliographic review

The original purpose of the bibliographic search differed from the current objectives of this study. We initially intended to estimate the correlation between the baseline and final measurements of the primary outcome. For this reason, the initial search was carried out with the aim of finding the variance of the difference between these two measurements in the article. Table S2 shows the specific search conducted for each year and the number of articles returned.

Table S2. Summary of the bibliographic review (previous) and the current search.These searches were run on the EBSCO version of MEDLINE. At present, we have tried to reproduce these searches via PubMed by adapting the syntax in a pertinent way, which resulted in a number of articles that were different from those retrieved previously. One explanation is that the factors may be different, such as the indexing of articles or the means of accessing the interfaces to Medline, among others.

	**Previous search (EBSCO)**			**Current search (PubMed)**		
**Year**	**no. papers**	**Search date**	**Search**	**no. papers**	**Search date**	**Search**
2004	266	January 2005	*AB(clinical trial* AND random*) AND AB (change OR evolution OR (difference AND baseline))*	*326*	*June 2017*	*(clinical trial*[Title/Abstract] AND random*[Title/Abstract]) AND (change[Title/Abstract] OR evolution[Title/Abstract] OR (difference[Title/Abstract] AND baseline[Title/Abstract])*
2007	348	January 2008		503	*June 2017*	
2010	478	January 2015	*AB((trial* AND random*) AND ((change OR evolution) AND*	*319*	*June 2017*	*(trial*[Title/Abstract] AND random*[Title/Abstract]) AND (change[Title/Abstract] OR evolution[Title/Abstract] AND*
2013	657	January 2015		442	*June 2017*	

			(difference AND baseline)))			*(difference[Title/Abstract] AND baseline[Title/Abstract]))*

One limitation of the search is that the terms "clinical trial" and "trial" were used instead of establishing a filter for this type of study. Although this limitation works against specificity (studies that were not clinical trials would be returned), it does not limit sensitivity (very few clinical trials omit these terms in the abstract). Therefore, the only inconvenience was the need for greater effort in filtering the articles found.

Section III: Descriptive measures

For reasons of interpretability, in this section we report the results using standard deviations (SD) instead of variances.

Table S3 contains the main statistics for the logarithm of the SD at the end and beginning of the study in both arms, as well as their differences. The log SD mean in the experimental arm at the end of the study (1.38) is lower than the same measure in the control group at the end of the study (1.41), and it is also lower than the log SD mean in the experimental group at the beginning of the study (1.44).

Table S3. Descriptive statistics for the logarithm of the SD (first four rows) and for their differences (last three rows). B: Baseline, O: Outcome, T: Treated or experimental, C: Control or reference. sd: standard deviation; min: minimum; Q1; first quartile; Q3: third quartile; max: maximum.

		n	mean	sd	min.	Q1	median	Q3	max.
**Baseline**	**Reference or Control arm (BC)**	208	1.38	1.61	-3.91	0.17	1.63	2.47	5.64
	**Experimental or Treated arm (BT)**	208	1.44	1.65	-3.51	0.14	1.80	2.60	6.83
**Final Outcome**	**Reference or Control arm (OC)**	208	1.41	1.63	-3.91	0.18	1.60	2.54	5.78
	**Experimental or Treated arm (OT)**	208	1.38	1.63	-3.51	0.10	1.51	2.51	6.73
**Differences**	**Between arms at the end (OT-OC)**	208	-0.06	0.38	-1.60	-0.17	-0.02	0.11	1.58
	**Over time in Experimental arm (OT-BT)**	208	-0.02	0.41	-2.15	-0.22	0.00	0.17	1.96
	**Over time between arms [(OT-BT) - (OC-BC)]**	208	-0.08	0.39	-1.47	-0.22	-0.05	0.06	2.55

Figure S1 to S4 highlight these differences for the comparison between arms and over-time, respectively, for each single study. As mentioned in the main body of the article, 113 (54.3%) studies have lower variability in the outcome at the end of the study in the experimental arm. On the other hand, 12 (5.8%) studies report exactly the same variability in both arms at the end of the study, and 83 (39.9%) provide a higher variability in the reference arm. For the over-time comparison in the treated arm, 97 (46.6%) interventions

increase the variability, 101 (48.6%) decrease it, and 11 (4.8%) do not affect the dispersion of the outcome.

Figure S1. Differences of standard deviations between arms. Arrow lengths are the differences in logarithms of the standard deviations between arms at the end of the study. Red arrows represent studies with greater variability in the control arm, and blue arrows in the treated. Y-axis is in log-scale.

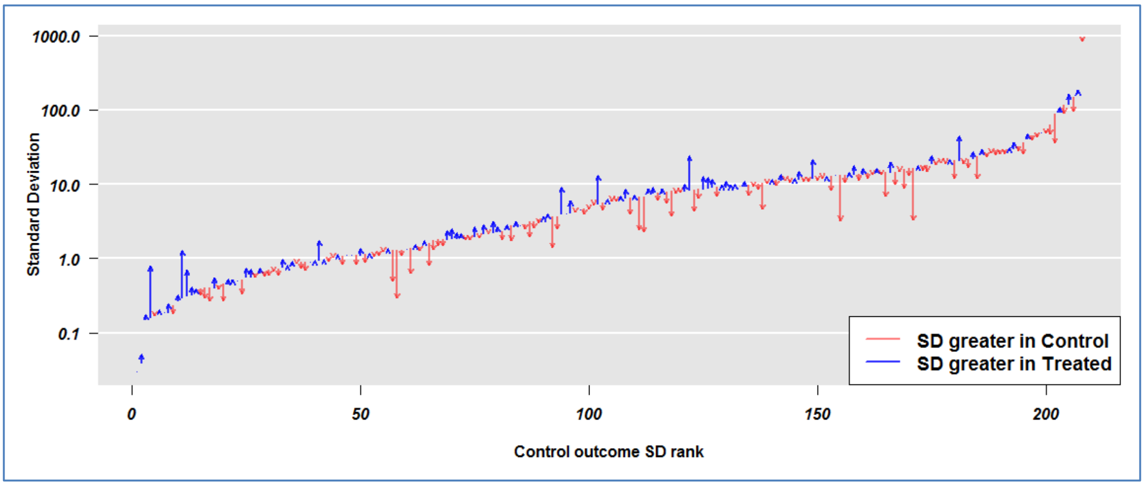

Figure S2. Histogram of the outcome standard deviations ratio between treated (OT) and control group (OC). Dashed vertical line represents the value for which both standard deviations are equal.

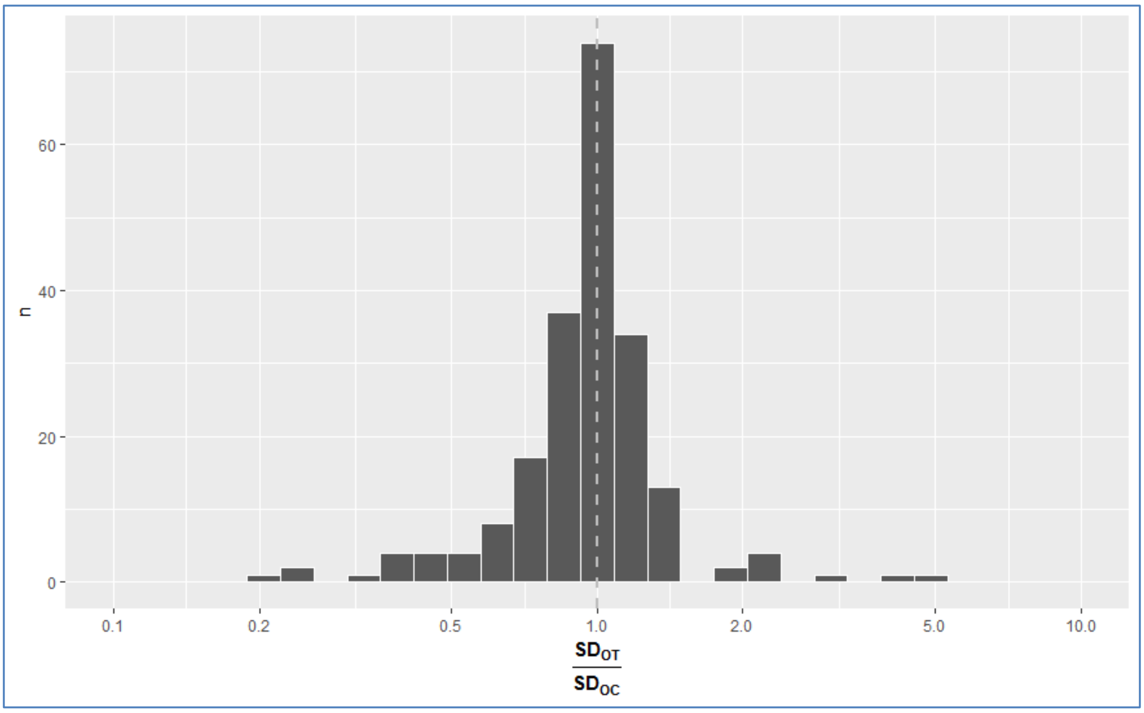

Figure S3. Differences of standard deviations over time. Arrow lengths are the differences in logarithms of the standard deviations between primary endpoint over time. Red arrows represent studies with greater variability at baseline, and blue arrows at the end of study. Y-axis is in log-scale.

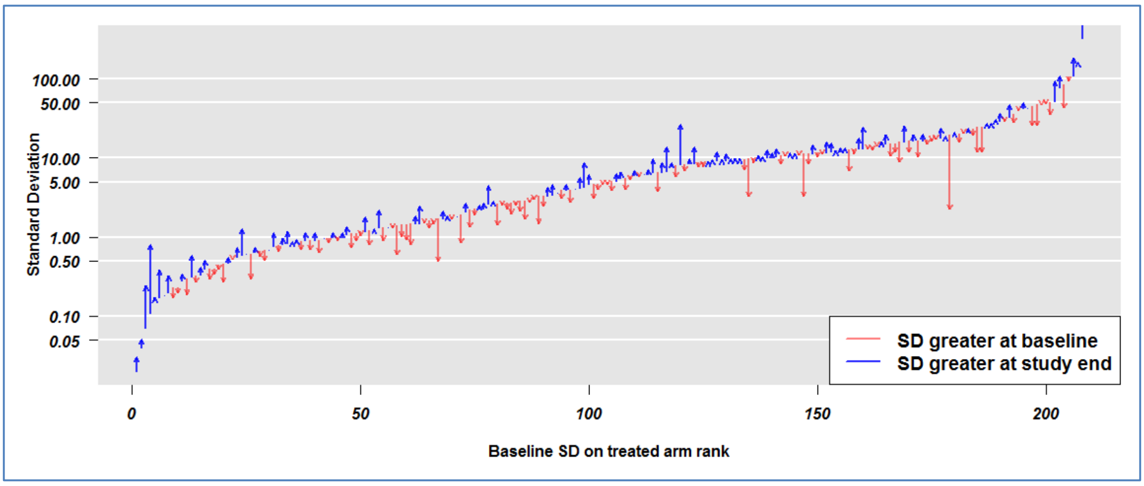

Figure S4. Histogram of the standard deviations ratio between final (OT) and baseline (BT) values in treated group. Dashed vertical line represents the value for which both standard deviations are equal.

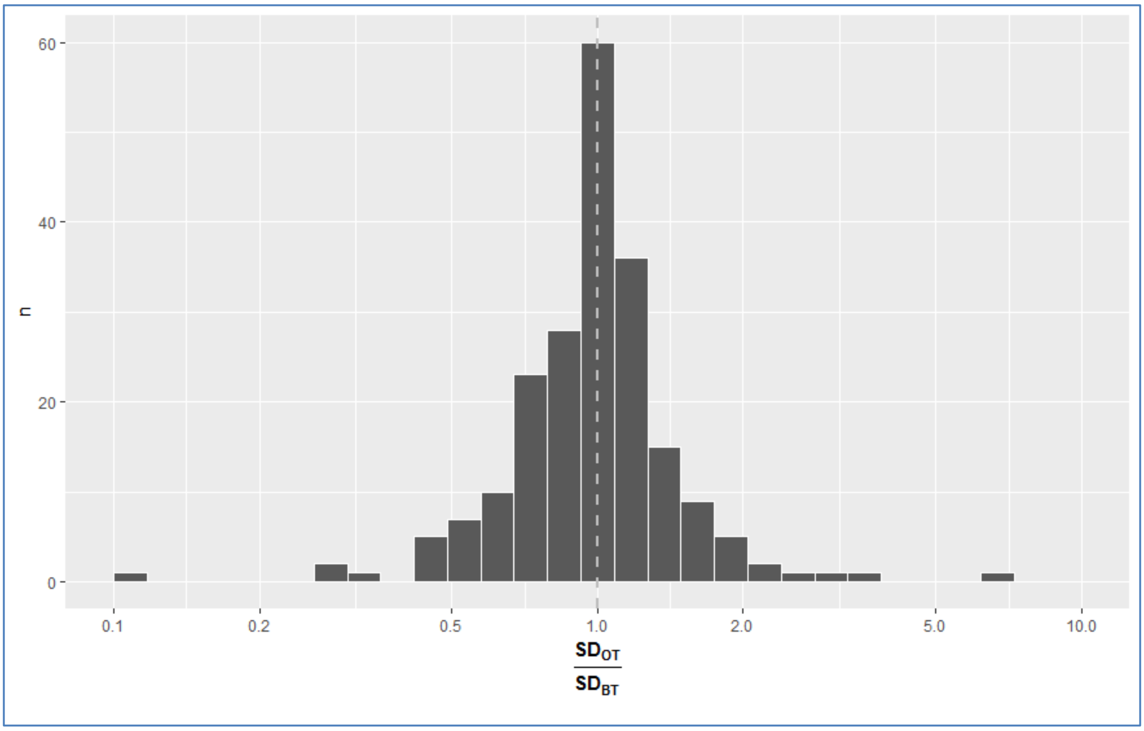

Section IV: Random-effects models

The fitted model for between-arm comparison was:

$$\log\left(\frac{V_{OT}}{V_{OC}}\right)_i = \mu + s_i + \beta \cdot \log\left(\frac{V_{BT}}{V_{BC}}\right)_i + e_i \quad with \quad e_i \sim N(0, v_i) \ and \ s_i \sim N(0, \tau)$$

V_{XX} represents the variances of the outcome in each arm at the end of the study (V_{OT}, V_{OC}) and at the baseline (V_{BT}, V_{BC}).The μ parameter is the expected response across all the studies; s_i represents the heterogeneity between-study effect with variance τ^2 associated to study i; β is the coefficient for the outcome measured at the beginning of the study; and e_i represents the within sample error with variance v^2.

An analogous model was employed to assess the homoscedasticity over time:

$$\log\left(\frac{V_{OT}}{V_{BT}}\right)_i = \mu + s_i + \beta \cdot \log\left(\frac{V_{OC}}{V_{BC}}\right)_i + e_i \quad with \quad e_i \sim N(0, v_i) \ and \ s_i \sim N(0, \tau)$$

We need to estimate v_i^2 for comparisons between arms and over time. For the former (independent samples), we approximated it using the *Delta* method as $2/df_T + 2/df_C$ (*24*), with df_T and df_C being the degrees of freedom of the samples in each arm. However, this expression underestimates the actual variance in small sample scenarios, and for this reason we set it to $2/(df_T-1) + 2/(df_C-1)$ in a similar way to what is done in the estimation of GEE models (11). We checked by simulation that this approach provided a better fit in the case of pure random variability (see Section VI for more information). For the over-time model (paired samples), we needed the covariance between the logarithm of variances (final versus initial), and this could be estimated only in the studies for which correlation between outcomes was available (see Section VII for more details)

Table S4. Estimated coefficients from the random mixed effects models. $\hat{\mu}$: Average of the main outcome among all the studies. $\hat{\beta}$: Coefficient for the main outcome measured at baseline (comparison between groups) or in control arm (comparison over time). $\hat{\tau}$: Standard deviation of the study random effect. The specifications of the models are:

[1] $\text{Log}(V_{BT}/V_{BC})_i = \mu + s_i + e_i \quad [s_i \sim N(0,\tau) \; is\ the\ study\ "effect"]$
[2] $\text{Log}(V_{OT}/V_{OC})_i = \mu + s_i + e_i$
[3] $\text{Log}(V_{OT}/V_{OC})_i = \mu + s_i + \beta_1 \cdot \log(V_{BT}/V_{BC})_i + e_i$
[4] $\text{Log}(V_{OT}/V_{OC})_i = \mu + s_i + 1 \cdot \log(V_{BT}/V_{BC})_i + e_i$
[5] $\text{Log}(V_{OT}/V_{BT})_i = \mu + s_i + e_i$
[6] $\text{Log}(V_{OT}/V_{BT})_i = \mu + s_i + \beta_1 \cdot \log(V_{OC}/V_{BC})_i + e_i$
[7] $\text{Log}(V_{OT}/V_{BT})_i = \mu + s_i + 1 \cdot \log(V_{OC}/V_{BC})_i + e_i$

The datasets used to fit these models are:
[A] **CDB**: **Complete Dataset for Between-arm comparison.** All 208 studies.
[B] **CDO**: **Complete Dataset for Over-time comparison.** 95 studies with enough information to perform this analysis (see Methods section)
[C] **RDBB**: **Reduced Dataset for Between-arm comparison at Baseline.** 4 excluded studies: those with absolute values that are 4 standard errors farther from the point of equality.
[D] **RDB**: **Reduced Dataset for Between-arm comparison.** 30 excluded studies: minimum number to exclude for obtaining heterogeneity similar using RDBB.
[E] **RDO**: **Reduced Dataset for Over-time comparison.** 22 excluded studies: minimum number to exclude for obtaining heterogeneity similar to reduced baseline data in over-time comparison.

**Response in the model**	**Type of model**	$\hat{\mu}$ **(95%CI)**	$\hat{\beta}$ **(95%CI)**	$\hat{\tau}$	
		Full Data (main analysis)	Full Data (main analysis)	Full Data (main analysis)	Reduced data (heuristic analysis)
Baseline variance ratio		**CDB[A] (n = 208)**	**CDB (n = 208)**	**CDB (n=208)**	**RDBB[C] (n=204)[8]**
	Reference model[1]	0.03 (-0.03, 0.09)	-	0.31	0.066
Outcome variance ratio		**CDB (n = 208)**	**CDB (n = 208)**	**CDB (n=208)**	**RDB[D] (n=178)[9]**
	Unadjusted[2]	-0.11 (-0.20, -0.01)	0	0.60	0.078
	Adjusted[3]	-0.12 (-0.21, -0.03)	0.48 (0.30, 0.65)	0.55	0.075
	Adjusted (offset)[4]	-0.14 (-0.24, -0.04)	1	0.60	0.189
Outcome vs. baseline ratio in treated group		**CDO (n = 95)**	**CDO[B] (n = 95)**	**CDO (n=95)**	**RDO[E] (n=63)[10]**
	Unadjusted[5]	-0.14 (-0.30, 0.01)	0	0.71	0.068
	Adjusted[6]	-0.15 (-0.28, -0.02)	0.63 (0.42, 0.84)	0.59	0.036
	Adjusted (offset)[7]	-0.16 (-0.29, -0.02)	1	0.62	0.285

The estimated parameters of the models for the variability comparisons between arms and over time are given in Table S4. First, looking at the central parameter (μ), baseline variances were balanced, which is in accordance with random allocation. Second, significantly negative estimates in both models for the outcome variance ratio suggest greater variability in the control arm on average, which is contrary to the expected results under the hypothesis that treatment interactions would increase variability in the treated arm. Third, τ represents the amount of additional heterogeneity not belonging to the theoretical variability due to random allocation. In the model with baseline as the response, this heterogeneity $\hat{\tau}$ is 0.31. As only methodological flaws can explain this extra-variability or heterogeneity – as John Carlisle stated in a recent paper (12) – we also studied how many studies had to be excluded to reduce it to a reasonable amount; thus, it was reduced to 0.07 when 4 extreme studies were removed. For example, the study by Hsieh et al. (13) adjusted for "baseline" values, but those were obtained 1 month after the treatment started. When we model the outcome variance ratio as response instead of the baseline ratio, heterogeneity is approximately doubled, irrespective of the way the model accounts for the baseline variance imbalance within any study. We needed to exclude 30 studies to lower this heterogeneity in the reduced data to 0.08, with 11 (5.3% over all studies) being in the line of a treatment effect with increasing variance and 19 (9.1%) leading to a reduction.

Section V: Subgroup analyses

We collected some features of the studies: whether or not the comparison for the primary outcome was significant (*No*/*Yes*); the experimental intervention type (*Non-pharmacological*/*Pharmacological*); the outcome type (*Scored* if a measurement scale was used or *Measured* if the endpoint had units of measure); the nature of the illness (*Chronic* or *Acute*); the measurement type (*Automatic* if it was provided directly by a device or *Assessed* if an evaluator was involved); and the improvement direction (*upwards* if higher value in the response implies better condition or *downwards*, otherwise).

For each subgroup and for the overall sample, Figure S5 shows the CI95% of the outcome variance ratio between arms. It can be observed that there are no great discrepancies in any subgroup. However, when we look at the significance in the main outcome, studies without effects are, on average, centered almost completely around equal variances, while those studies with an effect seem, on average, to have even less variability in the experimental arm. This point suggests that if the treatment has an effect on the mean, it also has an effect on diminishing the variance, which is in accordance with some kind of stabilizing effect. On the other hand, a trend can also be observed in studies with assessed measurement: they have less variability in the experimental group compared to the reference arm.

Figure S5. 95% confidence intervals for subgroup analyses on between-arm comparisons. For all data and each subgroup, the figure represents the 95% confidence intervals for the estimated outcome (O) variance ratio between Treated (T) and Controls (C), after being adjusted by baseline discrepancies through the *rma* function (Model 3 of Table S4). X-axis is in log scale.
*32 studies were performed with healthy participants, i.e., those without any particular illness.

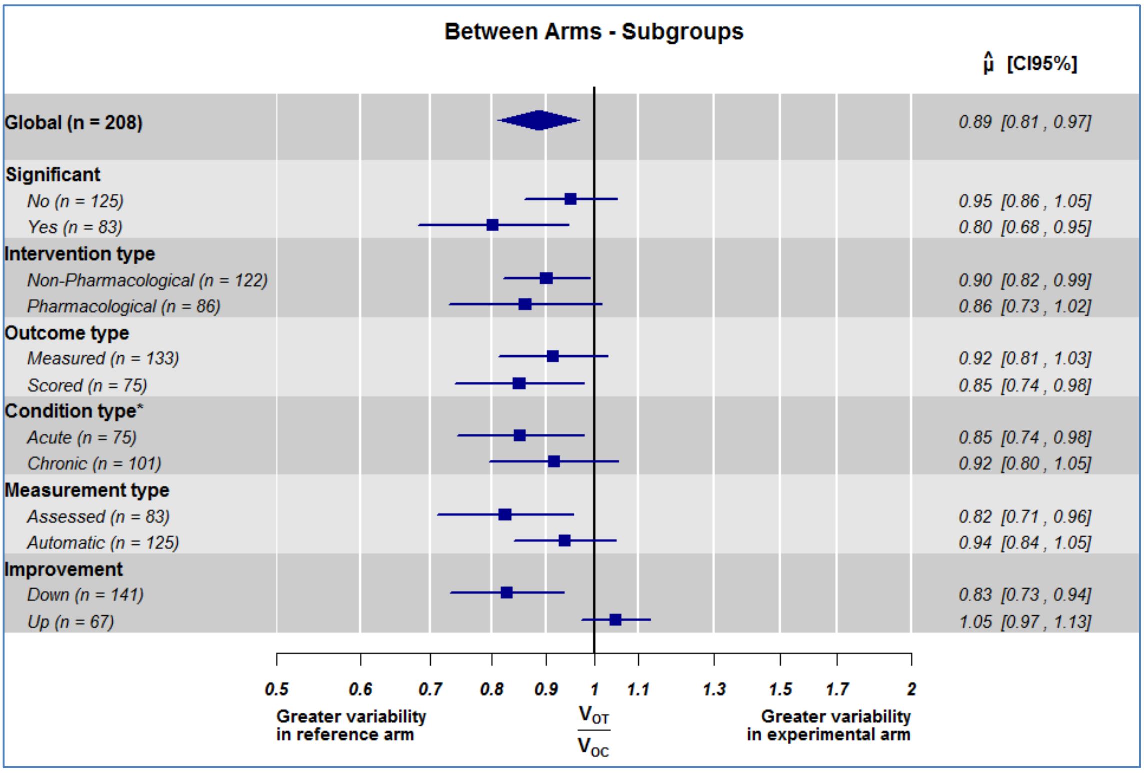

Figure S6 shows the CI95% for the over-time variance ratio in the experimental arm, and Figure S7 shows the difference between this ratio and the same response in the control group. No major additional conclusions arise from these results.

Figure S6. 95% confidence intervals for subgroup analyses on over-time comparisons (95 patients). For all Treated (T) arms and each subgroup within these arms, the figure represents the 95% confidence intervals for the estimated variance ratio between Outcome (O) and Baseline (B), after being adjusted by change over time ratio in the control group through the *rma* function (Model 6 of Table S4). X-axis is in log scale. *13 studies were performed with healthy participants, i.e., those without any particular illness.

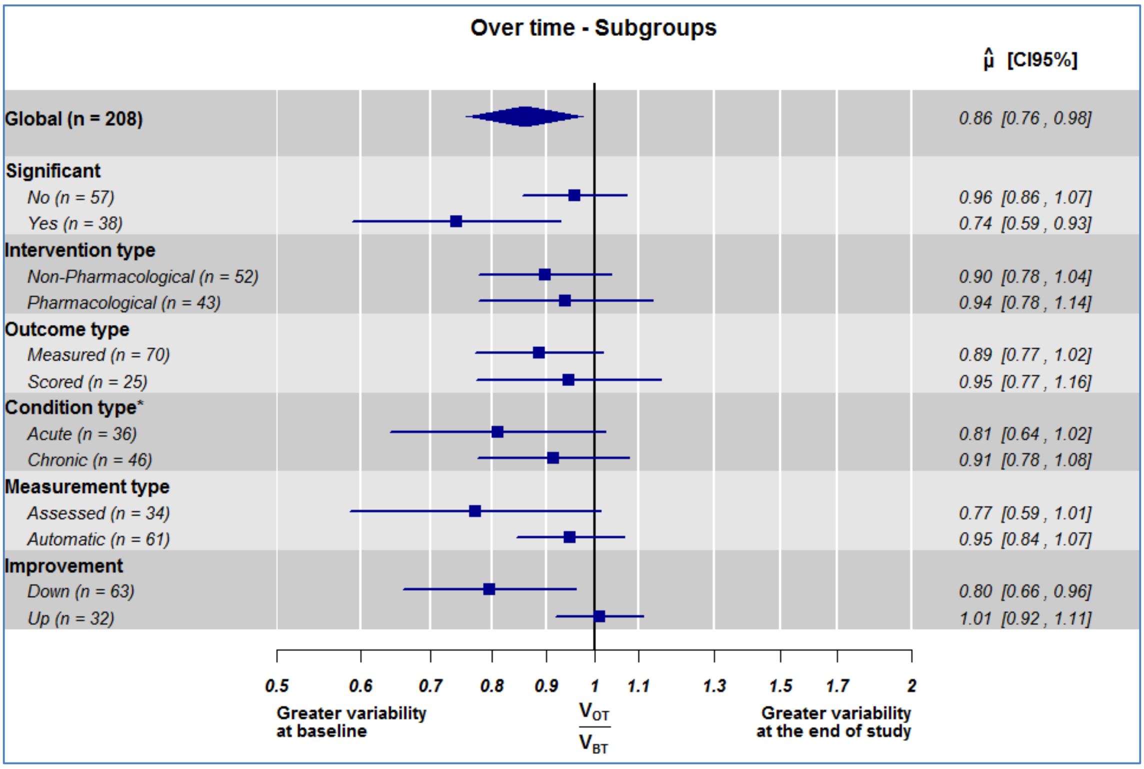

Figure S7. 95% confidence intervals for subgroup analyses on over-time comparison between arms. For all data and each subgroup, the figure represents the 95% confidence intervals for the estimated outcome (O) variance ratio between Treated (T) and Controls (C) (Model 7 of Table S4). X-axis is in log scale. *32 studies were performed with healthy participants, i.e., those without any particular illness.

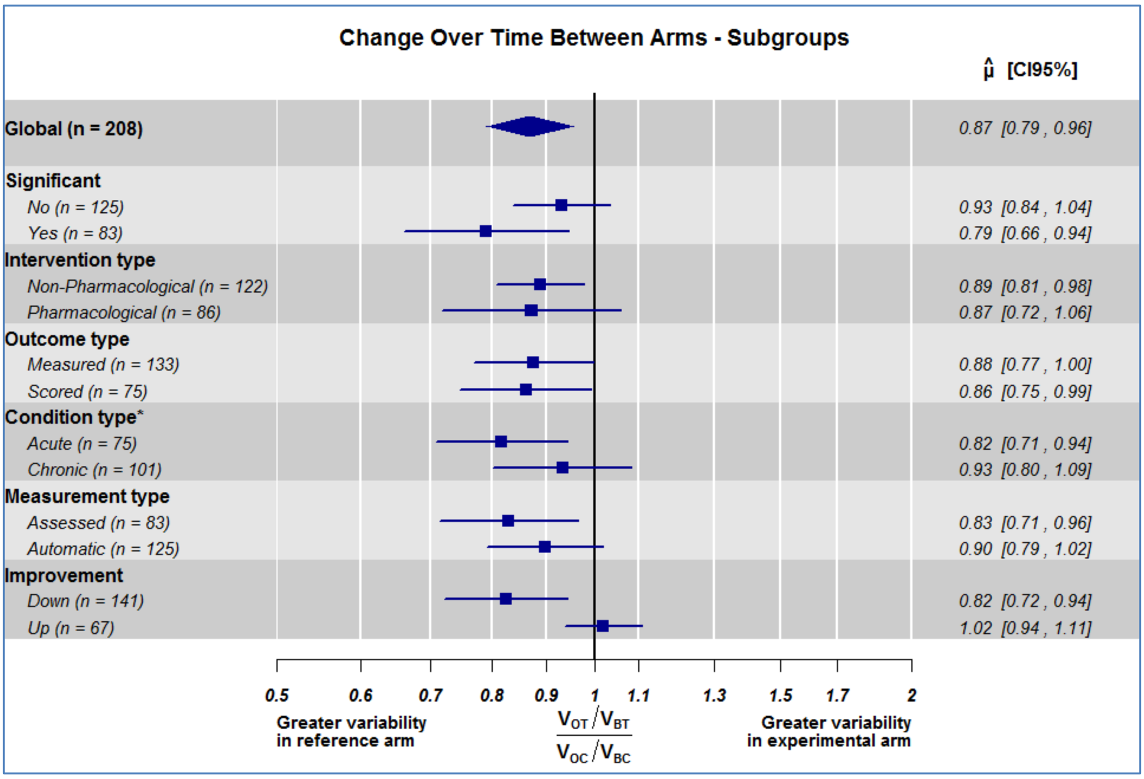

Section VI: Standard error of *log(V_{OT}/V_{OC})* in independent samples

Methodology

Through the delta method (DM), we derive the standard error (SE) of the logarithm of the variance as:

$$\sqrt{\frac{2}{df}}$$

Where *df* represents the degrees of freedom, i.e., the sample size minus 1. Therefore, the approximate SE of the logarithm of the variance ratio between two independent variances is:

$$\sqrt{\frac{2}{df_1}+\frac{2}{df_2}}$$

We performed a simulation process in order to obtain 2 different estimates (E1 and E2) of this SE. To do so, we first compared each one with the previous estimate (DM), and then again using the same statistic but with a correction in the denominator: *df* → *df-1*, which we named DMc (Delta Method corrected).

The parameters of the simulation were:

- 10,000 replications
- Actual variance of the first group (V1): 1 (constant)
- Actual variance of the second group (V2): 1 (constant)
- Sample size in first group (n1): 30, 300, 3000
- Sample size in second group (n2): n1/3 ; n1/2 ; n1

Estimation 1

For each iteration:

1. We simulate two samples from a Normal distribution of sample sizes n1 and n2 with mean 0 (in both cases) and variances V1=V2=1.
2. For each sample, we calculate its variance. We obtain v1 and v2.
3. We calculate the statistic log(v1/v2).

The first estimate (E1) was the standard deviation of all statistics obtained from the simulations

Estimation 2

For each iteration:

1. We simulate two values from two chi-square distributions with (n1-1) and (n2-1) degrees of freedom, respectively.
2. We multiply these two values by V1/(n1-1) and V2/(n2-1), respectively. We obtain v1 and v2.
3. We calculate the statistic log(v1/v2).

The second estimate (E2) was the standard deviation of all statistics obtained from the simulations

Results

Table S5 shows the results of the simulation. Each row represents a different scenario according to the sample size. Column "DM" represents the theoretical value obtained from the first formula presented in this section. Columns "E1" and "E2" represent the two estimates for each scenario. Finally, the columns "E1/DM" and "E2/DM" represent the ratio between the two estimates and that obtained with the delta method; Columns

“E1/DMc” and “E2/DMc” are the same ratios, but by using the correction mentioned above.

Table S5. Results from the simulation comparing different estimators for the standard error. Discrepancies with DMc are lower than those obtained with DM.

**Sample size**		**Standard Errors**							
**n1**	**n2**	**DM**	**DMc**	**E1**	**E1/DM**	**E1/DMc**	**E2**	**E2/DM**	**E2/DMc**
30	10	0.540	0.567	0.564	1.046	0.996	0.569	1.054	1.003
30	15	0.460	0.475	0.478	1.039	1.007	0.479	1.042	1.010
30	30	0.371	0.378	0.380	1.023	1.005	0.378	1.018	1.000
300	100	0.164	0.165	0.164	0.997	0.993	0.163	0.992	0.988
300	150	0.142	0.142	0.144	1.012	1.010	0.143	1.007	1.004
300	300	0.116	0.116	0.116	1.007	1.005	0.115	0.995	0.994
3000	1000	0.052	0.052	0.052	0.999	0.999	0.051	0.996	0.996
3000	1500	0.045	0.045	0.045	1.005	1.005	0.045	0.999	0.999
3000	3000	0.037	0.037	0.036	0.997	0.997	0.036	0.996	0.996

With large sample sizes (n1≥300), the relative differences were always less than 1% in all cases. For DM estimates, these differences were higher with small sample sizes (n1=30), having obtained a relative difference from 2% (in the scenario of equal sample sizes) to 5% (in the scenario where n2=n1/3). However, these discrepancies were reduced when we applied the correction to the delta method. In those cases, they are lower than 1% in all scenarios.

Section VII: Standard error of *log(V_{OT}/V_{BT})* in paired samples

In order to estimate the variance of the logarithm of the ratio for the paired cases, we need to estimate the covariance between the logarithms of the variances.

$$V\left[log\left(\frac{V_{OT}}{V_{BT}}\right)\right] = V[\log(V_{OT}) - log(V_{BT})]$$

$$= V[\log(V_{OT})] + V[\log(V_{BT})] - 2 \cdot Cov[\log(V_{OT}), \log(V_{BT})]$$

Let us recall that the variance of the logarithm of the variance can be estimated as follows, according to the delta method:

$$V[\log(V_{OT})] = \frac{2}{df_{OT}} \qquad V[\log(V_{BT})] = \frac{2}{df_{OB}}$$

Now we are going to show how to estimate the covariance.

<u>Step 1</u>. Demonstrate:

$$\boldsymbol{Cov[\log(V_{OT}), \log(V_{BT})] \approx \log\left[1 + \frac{Cov[V_{OT}, V_{BT}]}{E[V_{OT}] \cdot E[V_{BT}]}\right]}$$

Let

$U = \log(V_{OT})$ $V = \log(V_{BT})W = U + V$

$X = \exp(U)$ $Y = \exp(V)$

We use:

A covariance property → $Cov(X, Y) = E(XY) - E(X)E(Y)$

Taylor expansion → $E\big(exp(U)\big) \approx \exp(\mu_U) + \frac{\exp(\mu_U)}{2}\sigma_U^2 \approx \exp\left(\mu_U + \frac{\sigma_U^2}{2}\right)$

[The last approximation is exact for U Normal and good enough for $\sigma_U^2/2$, which is not too large for departures from Normal]

We can express the expected value for the X, Y product as follows:

$$E(XY) = E(\exp(U) \cdot \exp(V)) = E(exp(W)) \approx \exp\left(\mu_W + \frac{\sigma_W^2}{2}\right)$$

And we can express the variance of W as:

$$Var(W) = Var(U) + Var(V) + 2 \cdot Cov(U,V)$$

Finally, we can deduce an expression for the exponential of the covariance:

$$1 + \frac{Cov(X,Y)}{E(X) \cdot E(Y)} = \frac{E(XY)}{E(X) \cdot E(Y)} = \frac{\exp\left(\mu_W + \frac{\sigma_W^2}{2}\right)}{\exp\left(\mu_U + \frac{\sigma_U^2}{2}\right) \cdot \exp\left(\mu_V + \frac{\sigma_V^2}{2}\right)}$$

$$= \frac{\exp\left(\mu_U + \mu_V + \frac{1}{2} \cdot (\sigma_U^2 + \sigma_V^2 + 2 \cdot Cov(U,V))\right)}{\exp\left(\mu_U + \frac{\sigma_U^2}{2}\right) \cdot \exp\left(\mu_V + \frac{\sigma_V^2}{2}\right)} \approx exp[Cov(U,V)]$$

Therefore:

$$Cov(U,V) \approx log\left(1 + \frac{Cov(X,Y)}{E(X) \cdot E(Y)}\right) \rightarrow Cov[\log(V_{OT}), \log(V_{BT})]$$

$$= \log\left[1 + \frac{Cov[V_{OT}, V_{BT}]}{E[V_{OT}] \cdot E[V_{BT}]}\right]$$

<u>Step 2</u>. Demonstrate:

$$\boldsymbol{E[V_{OX}] \approx \frac{n_{OX}}{df_{OX}} \cdot \hat{V}_{OX}}$$

As we know:

$$\frac{(n-1) \cdot S^2}{\sigma^2} \sim \chi_{n-1}^2$$

We use this distribution to estimate the expected value for the sample variance:

$$E\left[\frac{(n-1) \cdot S^2}{\sigma^2}\right] = E[\chi_{n-1}^2] \rightarrow E[S^2] = \frac{\sigma^2}{n-1} \cdot E[\chi_{n-1}^2] = \frac{\sigma^2}{n-1} \cdot (n-1) = \sigma^2$$

$$\approx \frac{n}{n-1} \cdot S^2$$

Therefore:

$$\log\left[1+\frac{Cov[V_{OT},V_{BT}]}{E[V_{OT}]\cdot E[V_{BT}]}\right]=\log\left[1+\frac{Corr[V_{OT},V_{BT}]\cdot\sqrt{V[V_{OT}]\cdot V[V_{BT}]}}{\left(\frac{n}{df}\right)^2\cdot\widehat{V_{OT}}\cdot\widehat{V_{BT}}}\right]$$

Step 3. Demonstrate:

$$\boldsymbol{Corr[V_{OT},V_{BT}]\approx Corr[Y_{OT},Y_{BT}]^2}$$

That is, the square of the correlation between variances is a good enough approximation of the correlation between the variance of the outcomes, as can be observed in the simulation result presented in Figure S8.

Figure S8. Correlation between sample variances as a function of the correlation between outcomes resulting from a Normal data simulation. The red line represents the function $Y=X^2$ and the green line represents a non-parametric curve obtained from a locally-weighted polynomial regression (LOESS), which is close.

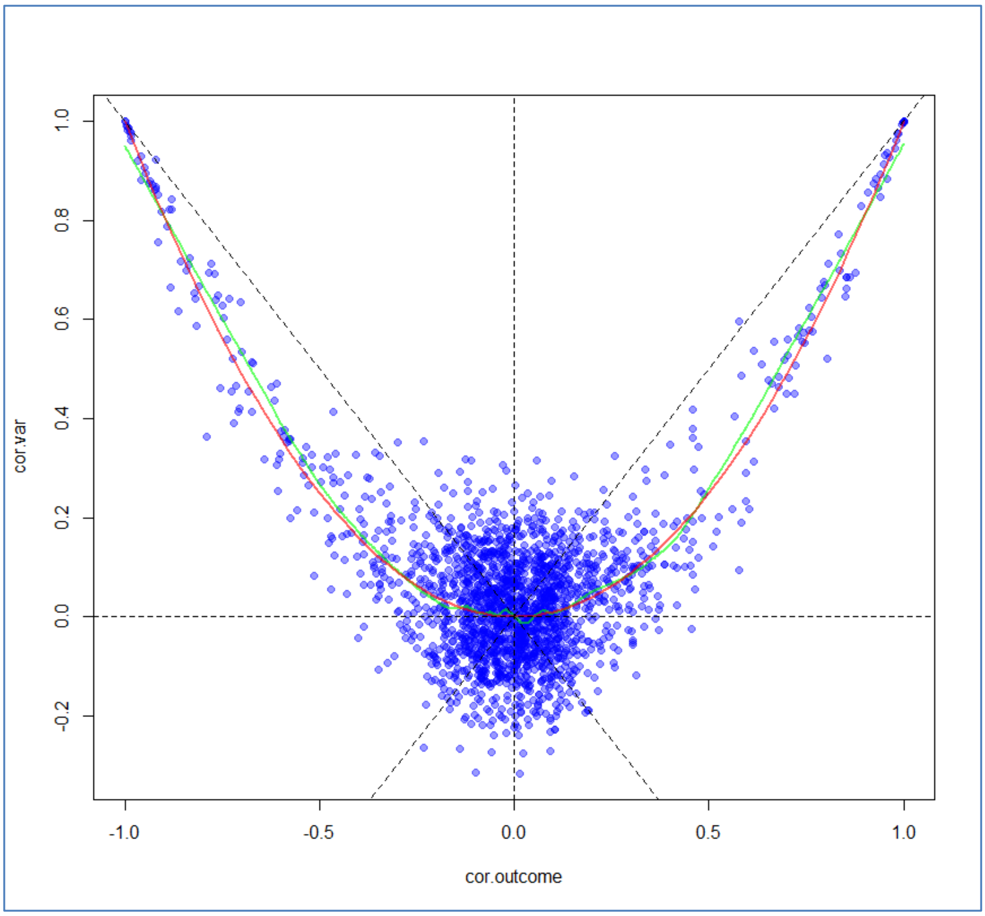

Therefore,

$$\log\left[1+\frac{Corr[V_{OT},V_{BT}]\cdot\sqrt{V[V_{OT}]\cdot V[V_{BT}]}}{\left(\frac{n}{df}\right)^2\cdot\widehat{V_{OT}}\cdot\widehat{V_{BT}}}\right]=\log\left[1+\frac{Corr[Y_{OT},Y_{BT}]^2\cdot\sqrt{V[V_{OT}]\cdot V[V_{BT}]}}{\left(\frac{n}{df}\right)^2\cdot\widehat{V_{OT}}\cdot\widehat{V_{BT}}}\right]$$

Step 4. Demonstrate:

$$\boldsymbol{V[V_{OX}]\approx\frac{2\cdot\hat{V}_{OX}^2}{df_{OX}}}$$

As we have mentioned above:

$$\frac{(n-1)\cdot S^2}{\sigma^2}\sim\chi_{n-1}^2$$

Then:

$$V\left[\frac{(n-1)\cdot S^2}{\sigma^2}\right]=V[\chi_{n-1}^2]\rightarrow V[S^2]=\frac{\sigma^4}{(n-1)^2}\cdot V[\chi_{n-1}^2]=\frac{\sigma^4}{(n-1)^2}\cdot V[\chi_{n-1}^2]$$

$$=\frac{\sigma^4}{(n-1)^2}\cdot 2\cdot(n-1)=\frac{2\sigma^4}{n-1}\approx\frac{2\cdot S^4}{df}$$

Therefore,

$$\log\left[1+\frac{Corr[V_{OT},V_{BT}]\cdot\sqrt{V[V_{OT}]\cdot V[V_{BT}]}}{\left(\frac{n}{df}\right)^2\cdot\widehat{V_{OT}}\cdot\widehat{V_{BT}}}\right]$$

$$=\log\left[1+\frac{Corr[Y_{OT},Y_{BT}]^2\cdot\sqrt{\frac{2\hat{V}_{OT}^2}{df}\cdot\frac{2\hat{V}_{BT}^2}{df}}}{\left(\frac{n}{df}\right)^2\cdot\widehat{V_{OT}}\cdot\widehat{V_{BT}}}\right]$$

$$=\log\left[1+\frac{Corr[Y_{OT},Y_{BT}]^2\cdot\frac{2\hat{V}_{OT}\cdot\hat{V}_{BT}}{df}}{\left(\frac{n}{df}\right)^2\cdot\widehat{V_{OT}}\cdot\widehat{V_{BT}}}\right]$$

$$=\boldsymbol{\log\left[1+\frac{2\cdot Corr[Y_{OT},Y_{BT}]^2}{n^2/df}\right]}$$

Finally, we have achieved an expression for estimating the variance of the log variance ratio as follows:

$$V\left[log\left(\frac{V_{OT}}{V_{BT}}\right)\right] = \frac{2}{df_{OT}} + \frac{2}{df_{BT}} - 2 \cdot \log\left[1 + \frac{2 \cdot Corr[Y_{OT}, Y_{BT}]^2}{n^2/df}\right]$$

Section VIII: Conditions for homoscedasticity to hold without a constant effect under an additive model

Let $Y(0)$ and $Y(1)$ be the potential outcomes in the reference and experimental groups, respectively. Under an additive model, $Y(1) = Y(0) + effect$, we can devise four situations:

- First, if we have a fixed effect model, then $V[effect] = 0$ and homoscedasticity holds.
- Second, a random effects model with no correlation. As $V[effect]$ cannot be negative, $V[Y(1)]$ should be larger than $V[Y(0)]$, implying heteroscedasticity.
- Third, a random effects model with positive correlation. This situation also leads to heteroscedasticity, with $V[Y(1)] > V[Y(0)]$.
- And fourth, a random effects model with a negative correlation...:
    - ... equal to $-\frac{1}{2} \cdot \frac{\sigma_{effect}}{\sigma_{Y(0)}}$, then $V[Y(1)] = V[Y(0)]$;
    - ... higher than this value, then $V[Y(1)] > V[Y(0)]$; and
    - ... lower than this value, then $V[Y(1)] < V[Y(0)]$.

So, under the standard parametrization of an additive effect, the only situation without a constant effect which leads to homoscedasticity has a specific negative correlation. For example, if the variability of the effect equals the natural variability of the outcome under the control treatment, this negative correlation should be -0.5. However, if the variability of the effect is smaller (say 10% of $\sigma_{Y(0)}$), which is a nearly constant treatment effect at a practical level, this correlation should be less than -0.05.

In any case, we have already shown in Figure 4 that homoscedasticity does not imply a constant effect. Critics of medical protocols developed through classical evidence-based

medicine may argue that ATE does not imply a constant effect. However, we propose that some evidence is needed to indicate that the effect is not constant before advocating for precision medicine (rather than the opposite).

In summary, medical researchers must be transparent about their premise; and statisticians should develop methods to study these premises

References

1. Graham HR, Ayede AI, Bakare AA, et al. Improving oxygen therapy for children and neonates in secondary hospitals in Nigeria: study protocol for a stepped-wedge cluster randomised trial. Trials [Internet] 2017;18(1):502. Available from: http://trialsjournal.biomedcentral.com/articles/10.1186/s13063-017-2241-8
2. Barone MA, Mbuguni Z, Achola JO, et al. Safety of tubal ligation by minilaparotomy provided by clinical officers versus assistant medical officers: study protocol for a noninferiority randomized controlled trial in Tanzanian women. Trials [Internet] 2017;18(1):499. Available from: http://trialsjournal.biomedcentral.com/articles/10.1186/s13063-017-2235-6
3. Park J-B, Jung J-H, Yoon YE, et al. Long-term Effects of high-doSe pitavaStatin on Diabetogenicity in comparison with atorvastatin in patients with Metabolic syndrome (LESS-DM): study protocol for a randomized controlled trial. Trials [Internet] 2017;18(1):501. Available from: http://trialsjournal.biomedcentral.com/articles/10.1186/s13063-017-2229-4
4. Wu W, Deng H, Rao N, et al. Neoadjuvant everolimus plus letrozole versus fluorouracil, epirubicin and cyclophosphamide for ER-positive, HER2-negative breast cancer: study protocol for a randomized pilot trial. Trials [Internet] 2017;18(1):497. Available from: http://trialsjournal.biomedcentral.com/articles/10.1186/s13063-017-2228-5
5. Shepperd S, Cradduck-Bamford A, Butler C, et al. A multi-centre randomised trial to compare the effectiveness of geriatrician-led admission avoidance hospital at home versus inpatient admission. Trials [Internet] 2017;18(1):491. Available from:

http://trialsjournal.biomedcentral.com/articles/10.1186/s13063-017-2214-y%0Ahttp://www.ncbi.nlm.nih.gov/pubmed/29061154

6. Jakkula P, Reinikainen M, Hästbacka J, et al. Targeting low- or high-normal Carbon dioxide, Oxygen, and Mean arterial pressure After Cardiac Arrest and REsuscitation: study protocol for a randomized pilot trial. Trials [Internet] 2017;18(1):507. Available from: http://www.ncbi.nlm.nih.gov/pubmed/29084585%0Ahttp://trialsjournal.biomedcentral.com/articles/10.1186/s13063-017-2257-0
7. Salaminios G, Duffy L, Ades A, et al. A randomised controlled trial assessing the severity and duration of depressive symptoms associated with a clinically significant response to sertraline versus placebo, in people presenting to primary care with depression (PANDA trial): study protocol for a randomised controlled trial. Trials [Internet] 2017;18(1):496. Available from: http://trialsjournal.biomedcentral.com/articles/10.1186/s13063-017-2253-4
8. Gal R, Monninkhof EM, Groenwold RHH, et al. The effects of exercise on the quality of life of patients with breast cancer (the UMBRELLA Fit study): study protocol for a randomized controlled trial. Trials [Internet] 2017;18(1):504. Available from: http://trialsjournal.biomedcentral.com/articles/10.1186/s13063-017-2252-5
9. Tamura T, Hayashida K, Sano M, Onuki S, Suzuki M. Efficacy of inhaled HYdrogen on neurological outcome following BRain Ischemia During post-cardiac arrest care (HYBRID II trial): study protocol for a randomized controlled trial. Trials [Internet] 2017;18(1):488. Available from: http://trialsjournal.biomedcentral.com/articles/10.1186/s13063-017-2246-3

10. Jefford M, Emery J, Grunfeld E, et al. SCORE: Shared care of Colorectal cancer survivors: protocol for a randomised controlled trial. Trials [Internet] 2017;18(1):506. Available from: http://trialsjournal.biomedcentral.com/articles/10.1186/s13063-017-2245-4
11. Mancl LA, DeRouen TA. A covariance estimator for GEE with improved small-sample properties. Biometrics, 2001;57:126-34.
12. Carlisle J. Data fabrication and other reasons for non-random sampling in 5087 randomised, controlled trials in anaesthetic and general medical journals. Anaesthesia, 2017;72:10.1111/anae.13938.
13. Hsieh LL, Kuo CH, Yen MF, Chen TH. A randomized controlled clinical trial for low back pain treated by acupressure and physical therapy. Prev. Med, 2004;39:168-76.
