# Supplementary material for: Does evidence support the high expectations placed in precision medicine? A bibliographic review
Source: F1000Res. 2019 Jun 10;7:30. Originally published 2018 Jan 9. [Version 5] doi: 10.12688/f1000research.13490.5 (PMC6524747; doi:10.12688/f1000research.13490.5)
Supplement: Peer review history

# Peer review: Reviewer response for version 5

**DOI:** 10.5256/f1000research.21429.r49728

**Published:** 2019-06-12

**Authors:** Vance W. Berger

**Affiliations:** 1 National Cancer Institute, Rockville, MD, USA

I have read this submission. I believe that I have an appropriate level of expertise to confirm that it is of an acceptable scientific standard.

# Peer review: Reviewer response for version 4

**DOI:** 10.5256/f1000research.20226.r47666

**Published:** 2019-05-15

**Authors:** Dennis W. Lendrem

**Affiliations:** 1 Musculoskeletal Research Group, Institute of Cellular Medicine, Newcastle University, Newcastle upon Tyne, UK; 2 NIHR Newcastle Biomedical Research Centre, Newcastle University, Newcastle upon Tyne, UK

This paper makes an important point and goes some way to tempering the high expectations placed on precision medicine. 

 Have the authors shown that the comparison of variances is a useful but not definitive tool for assessing whether or not the assumption of a constant effect holds?  I think they have.

 Would more data be useful? I think it would. The paper could be misinterpreted as an attack on precision medicine. This is unfair.  In this version, the authors have tempered their conclusions in an appropriate manner. The study is based on just 208 of the eligible studies. It seems likely that studies permitting the analysis are a select subset of the studies available. And, given the uncertainties surrounding estimates of variance components, it seems not unreasonable to assume that not all studies were equally likely to have detected differences in outcome variance. However, the authors have defined a method and a process permitting evidence in support of precision medicine to be discussed rationally.

I have read this submission. I believe that I have an appropriate level of expertise to confirm that it is of an acceptable scientific standard.

# Response

**Published:** 2019-05-31

**Authors:** Jordi Cortés

**Affiliations:** Universitat Politècnica de Catalunya, Spain

This paper makes an important point and goes some way to tempering the high expectations placed on precision medicine.

**We are grateful to Professor Dennis Lendrem for his review and for his comments, which positively evaluate our article. Indeed, the message that we want to highlight in our work is that precision medicine can be useful under certain circumstances, but *there is still (a lot of) room for “generic” *medicine, and researchers must take into account that the study of variability in their data provides valuable information to all of society.**

Have the authors shown that the comparison of variances is a useful but not definitive tool for assessing whether or not the assumption of a constant effect holds?  I think they have.

**Thank you. We have shown some prudence in this statement because we know that there may be other techniques to evaluate the presence of a constant effect, such as the one proposed by Caughey et al. [1]**

1. **Caughey D, Dafoe A, Miratix L. Beyond the sharp null: permutation tests actually test heterogeneous effects. Summer meeting of the Society for Political Methodology; 21–23 July 2016; Rice University;**

Would more data be useful? I think it would. The paper could be misinterpreted as an attack on precision medicine. This is unfair.  In this version, the authors have tempered their conclusions in an appropriate manner. The study is based on just 208 of the eligible studies. It seems likely that studies permitting the analysis are a select subset of the studies available. And, given the uncertainties surrounding estimates of variance components, it seems not unreasonable to assume that not all studies were equally likely to have detected differences in outcome variance. However, the authors have defined a method and a process permitting evidence in support of precision medicine to be discussed rationally.

**Thank you. We completely agree. Our work focuses on a narrow spectrum of studies with a specific design (parallel randomized controlled trials) and with a numerical outcome. In addition, the lack of information in many studies due to poor reporting may suggest that the sample is not completely representative. For these reasons, we believe that more studies should be conducted around this topic.**

# Peer review: Reviewer response for version 4

**DOI:** 10.5256/f1000research.20226.r47668

**Published:** 2019-04-29

**Authors:** Vance W. Berger

**Affiliations:** 1 National Cancer Institute, Rockville, MD, USA

The methods involve only variability, yet the conclusions are about precision medicine. It is not entirely clear what the one has to do with the other. Yes, I get that heterogeneity can mess up the analysis of precision medicine, but it can also mess up standard analyses of standard treatments too.  Moreover, is this really the strongest argument against precision medicine? Because it does not seem to be insurmountable, so if anything, it seems to argue against the research methods used to evaluate precision medicine, rather than against precision medicine itself. Also, the writing is fairly poor, which is surprising, since Dr. Senn is one of the best writers I have ever had the pleasure of reading.

I have read this submission. I believe that I have an appropriate level of expertise to confirm that it is of an acceptable scientific standard, however I have significant reservations, as outlined above.

# Response

**Published:** 2019-05-31

**Authors:** Jordi Cortés

**Affiliations:** Universitat Politècnica de Catalunya, Spain

The methods involve only variability, yet the conclusions are about precision medicine. It is not entirely clear what the one has to do with the other. Yes, I get that heterogeneity can mess up the analysis of precision medicine, but it can also mess up standard analyses of standard treatments too.

**Many thanks for your comments that will help us to clarify our paper.**

**Yes, we have concentrated on the analysis of variability. As we show (e.g., figures 1, panels A and B) a constant effect of the intervention allows stakeholders to provide unique advice to patients within the eligibility criteria. And also (2 ^nd^ paragraph of the introduction), a constant effect implies equal variances among the treatment groups. So, our aim was to show readers that the comparison of variances could be a useful way to provide information about the need to further personalize the clinical advice.**

**In our article we deal with heteroscedasticity and heterogeneity. The former is the main objective of the study and it refers to the presence of different variances in the two treatment arms. The measure we used to quantify it was the ratio of variances between arms. The heterogeneity, however, refers to how this measure oscillates between the different included studies. The measure to assess it was the tau^2 statistic obtained from the random-effects model. As this referee points out, the high heterogeneity in the variance ratio across the studies indicates that we cannot establish a single conclusion for every single study. Our results highlight that heteroscedasticity is negligible in most of the published parallel trials with numerical outcome, and thus, precision medicine would seem unjustified.**

**As we argue in the discussion section, we do not want to end precision medicine; we simply advocate the rational use of it while assessing the costs and benefits of its implementation in each specific situation.**

Moreover, is this really the strongest argument against precision medicine? Because it does not seem to be insurmountable, so if anything, it seems to argue against the research methods used to evaluate precision medicine, rather than against precision medicine itself.

**Thanks for the appraisement. Yes, you are right; surely, this is not the definitive argument against precision medicine, but we believe it is a well-founded method (variance comparison) that every researcher could easily apply in their studies for assessing the constant effect assumption. We just want to emphasize that, in any sense, we have to develop and use methods to observe the convenience for precision medicine. As we mentioned in the previous point, we warn against abuse when there is no evidence that the treatment effect varies among patients.**

Also, the writing is fairly poor, which is surprising, since Dr. Senn is one of the best writers I have ever had the pleasure of reading.

**Of course, we share your opinion about Stephen Senn's writing skills. The three main authors (JC, JAG and EC) are not native English speakers. We have worked closely with Matthew Elmore, an author working both as English editor and patient representative. As a result of your comment, in this latest version we have done our best to really improve the readability of the manuscript and we have completely restructured the Discussion section to clarify the message.**

# Peer review: Reviewer response for version 4

**DOI:** 10.5256/f1000research.20226.r45563

**Published:** 2019-04-10

**Authors:** David Nunan, Richard Stevens

**Affiliations:** 1 University of Oxford, Oxford, UK; 2 University of Oxford, University of Oxford, Oxford, UK

We enjoyed reading the paper by Cortes and colleagues as it very usefully surveys the literature for evidence that personalised effects exist, and finds little. This well-conceived study has also been well-executed but does not currently meet the same quality of reporting.

 The authors also need to be careful not to overstate their case: they find the absence of evidence, not evidence of absence. Whilst, on the whole, they express this correctly, in a few places, a previous reviewer correctly challenges them on this.

 Regarding the verb 'need', the first reviewer is right - it is too much to say 'If the effect randomly varies ... we need to characterize its distribution'. The authors themselves clarify, later in their reply to this reviewer, that what they mean is 'the best clinicians would like to know ...'.  'need' is a much stronger word than 'would like'. The Oxford English Dictionary defines it in terms of 'necessary', which in turn is defined as 'Indispensable, vital, essential, requisite'.  

 Prof White is also correct to challenge the remark on page 9 that 'the simplest interpretation ... thus rendering futile any search ...'

 The problem here is that the current wording suggests futility follows from equal variances. We feel it needs some form of wording that makes it clear that the 'futile' is conditional on 'the simplest interpretation' being true and suggest '... treatment effect is constant, which, if correct, would render futile any ...'

 Additional areas for clarification include:

 Abstract conclusion - where the authors state ‘…outcome variance was more often smaller…’, we find the results in both the Abstract and main Results section do not fully support this conclusion. Table 1 indicates that the most common outcome by far is 'variability not changed'. The authors should re-write the Abstract in such a way that the results directly support the conclusions.

 Methods - regarding the target population, it could be made more clear that 'quantitative outcomes' refers to outcomes with continuous data.

 Results - the results section cites figures in the Supplementary material but the Table (Table 1) and Figure (Figure 3) included in the main paper are not discussed.  It is hard to map results in the Abstract to the corresponding points in the main Results and hard to map conclusions directly to results, especially within the Abstract.

 Figure 2 - the percentages in brackets are confusing. Reporting according to current reporting standards is recommended.

 Discussion: on page 9 [PDF] where the authors state ‘…thus in turn also supporting evidence-based medicine’ and later on page 10 'Here, we have provided empirical insights for the rationale behind Evidence-Based Medicine.'

 Both statements equate the entire philosophy and practice of evidence-based medicine/practice with 'let's use parallel group trials'. This paper deals with one element of EBM/P - that of treatment decisions and a further distillation still down to the use of parallel controlled trials to guide decision making in these circumstances. The authors should re-word these occurrences accordingly.

We have read this submission. We believe that we have an appropriate level of expertise to confirm that it is of an acceptable scientific standard, however we have significant reservations, as outlined above.

# Response

**Published:** 2019-05-31

**Authors:** Jordi Cortés

**Affiliations:** Universitat Politècnica de Catalunya, Spain

We enjoyed reading the paper by Cortes and colleagues as it very usefully surveys the literature for evidence that personalised effects exist, and finds little. This well-conceived study has also been well-executed but does not currently meet the same quality of reporting.

**We are grateful to Professor Richard Stevens and Professor David Nunan for their comments, which helped us to clarify/improve our manuscript.**

**Next, we answer their comments and specify the modifications introduced in the manuscript accordingly.**

The authors also need to be careful not to overstate their case: they find the absence of evidence, not evidence of absence. Whilst, on the whole, they express this correctly, in a few places, a previous reviewer correctly challenges them on this.

**That’s right. The correct message is exactly that: there is not enough evidence for wide use of personalized medicine, but some sentences still remained imprecise. We are grateful to the previous referees who alerted us to this fact.**

Regarding the verb 'need', the first reviewer is right - it is too much to say 'If the effect randomly varies we need to characterize its distribution'. The authors themselves clarify, later in their reply to this reviewer, that what they mean is 'the best clinicians would like to know ...'.  'need' is a much stronger word than 'would like'. The Oxford English Dictionary defines it in terms of 'necessary', which in turn is defined as 'Indispensable, vital, essential, requisite'. 

**Thank you. We tried to clarify this issue in response to Prof White. However, we want to emphasize that this sentence came from an answer to the referee; but it has never been included in the manuscript.**

**Anyway, in this new version, we have replaced the term "need" in almost every sentence where it appeared (1 of them in the Abstract) for other expressions in order to ease the message.**

Prof White is also correct to challenge the remark on page 9 that 'the simplest interpretation ... thus rendering futile any search ...' The problem here is that the current wording suggests futility follows from equal variances. We feel it needs some form of wording that makes it clear that the 'futile' is conditional on 'the simplest interpretation' being true and suggest '... treatment effect is constant, which, if correct, would render futile any ...'

**Thank you for this important insight. First, we have tried to soften the tone of the phrase by changing "the simplest interpretation" to "a simple and believable interpretation".**

**Second, we have reworded the last part of the sentence according to the reviewers’ suggestion:**

**“…treatment effect is constant, which, if correct, would render futile any search for predictors of differential response”**

**With these wording improvements, we have decided to move this sentences to the beginning of the Discussion.**

Additional areas for clarification include:

Abstract conclusion - where the authors state ‘…outcome variance was more often smaller…’, we find the results in both the Abstract and main Results section do not fully support this conclusion. Table 1 indicates that the most common outcome by far is 'variability not changed'. The authors should re-write the Abstract in such a way that the results directly support the conclusions.

**Thank you for your suggestion**. **We refer to merely descriptive results. However, to avoid ambiguities, we have rewritten the Abstract and the Results section:**

**Previous Abstract: *We found that the outcome variance was more often smaller in the intervention group,…***

**Current Abstract: *The mean variance ratio is significantly lower than 1 and the lower variance was found more often in the intervention group than in the control group, suggesting it is more usual for treated patients to be stable.***

**Previous Results: *On average, the outcome variance ratio is close to one, with evidence of smaller variability in the treated arm.***

**Current Results: *The mean variance ratio is significantly lower than 1 and the lower variance was found more often in the intervention group than in the control group.***

Methods - regarding the target population, it could be made more clear that 'quantitative outcomes' refers to outcomes with continuous data.

**Thank you for the comment.  In fact, our work includes both studies with continuous and discrete outcomes (e.g., scales). For this reason, we have changed the term "quantitative" to "numerical", which we believe best reflects the outcome type in the eligibility criteria.**

Results - the results section cites figures in the Supplementary material but the Table (Table 1) and Figure (Figure 3) included in the main paper are not discussed.  It is hard to map results in the Abstract to the corresponding points in the main Results and hard to map conclusions directly to results, especially within the Abstract.

**Thank you for this point. On the one hand, now, we have introduced Figure 3 earlier and we have included some sentences to explain it. On the other hand, we have repeated the citation of Table 1 in several places in order to clarify the source of each percentage showed in it.**

Figure 2 - the percentages in brackets are confusing. Reporting according to current reporting standards is recommended.

**Thank you for your comment. We have rewritten the percentages in Figure 2 according to the total number of initial documents obtained from the bibliographic search.**

Discussion: on page 9 [PDF] where the authors state ‘…thus in turn also supporting evidence-based medicine’ and later on page 10 'Here, we have provided empirical insights for the rationale behind Evidence-Based Medicine.' Both statements equate the entire philosophy and practice of evidence-based medicine/practice with 'let's use parallel group trials'. This paper deals with one element of EBM/P - that of treatment decisions and a further distillation still down to the use of parallel controlled trials to guide decision making in these circumstances. The authors should re-word these occurrences accordingly.

**We agree that the interpretation is overemphasized according to the findings of our work. We have tried to improve both sentences:**

**Previous sentence: *…thus in turn also supporting evidence-based medicine***

**Current sentence: *…thus in turn also supporting the use of parallel controlled trials to guide decision-making in these circumstances.***

**Previous sentence: *Here, we have provided empirical insights for the rationale behind Evidence-Based Medicine.***

**Current sentence: *Here, we have provided empirical insights to postulate that such a premise is reasonable in most published parallel clinical trials.***

# Peer review: Reviewer response for version 3

**DOI:** 10.5256/f1000research.17220.r40679

**Published:** 2018-12-19

**Authors:** Ian R. White

**Affiliations:** 1 Medical Research Council Clinical Trials Unit, Institute of Clinical Trials and Methodology, University College London, London, UK

I consider each of the points in turn.

 1. We are debating whether “conventional clinical trials are designed to find differences with the implicit assumption that the effect is the same in all patients within the eligibility criteria”.

 The authors quote Paul Holland. The passage quoted is a careful definition of the “constant effect assumption”, but it does not support the authors’ argument - it does not claim that this assumption is required in randomised trials. Indeed elsewhere in his paper, Holland points out the value of an average causal effect:

 “The value of the average causal effect T is of potential interest for its own sake in certain types of studies. It would be of interest to a state education director who wanted to know what reading program would be the best to give to all of the first graders in his state. The average causal effect of the best program would be reflected in increases in statewide average reading scores.”

 I entirely agree with the authors that

 “Without this [constant effect] assumption, the value of the “average causal effect” is not enough to convey all the information about the treatment effect.”

 but I don’t agree that

 “If the effect randomly varies among the different units (as shown in panel D of Figure 1), we need to characterize its distribution: for example, by a normal distribution with its mean (delta) and standard deviation (SD).”

 My disagreement is over the word “need”. It would be really useful  to characterize the distribution. But we usually can’t do that in RCTs (cross-over trials and n-of-1 trials being notable exceptions). If we can’t characterize the distribution, the mean is still useful, as in the reading program example above, or as in treatment of a group of patients. As I said in my original review:

 “This is why the trials community worries so much about external generalisability: for example, if a trial treated 60% women and 40% men and showed a benefit of treatment, then a clinician treating women and men in the same ratio can be confident of giving a benefit overall, but a clinician treating women and men in a different ratio cannot be so confident.”

 2. Here we are debating whether the authors’ conclusions follow validly from the observed reduction in variance in the treated arm. The authors have included plenty of caveats. But their primary statement (in the abstract) remains “treated patients … would not require further precision medicine”: I still disagree with this. Other over-statements in the discussion are

 “When both arms have equal variances, then the simplest interpretation is that the treatment effect is constant, thus rendering futile any search for predictors of differential response.” (p9)

 “For most trials, the variability of the response to treatment changes scarcely or even decreases, which suggests that precision medicine’s scope may be less than what is commonly assumed” (p10)

 “There is evidence of effect variation in around 1 out of 7 trials, suggesting a limited role for tailored interventions” (p10)

 3. I find the presentation of the methods and results clearer now, though some parts remain poorly explained:

- In the formula for I^2, nu^2 is not the expected value of the error variance, since the latter is not a random variable.
- The formulae for V[log(V_OT/V_OC)] and V[log(V_OT/V_BT)] are in fact formulae for their within-study variances nu_i^2: this should be made clear. As written, they wrongly appear to be the total variances, which also involve tau^2
- In Table 1, “Random model” is an inadequate description of the complex procedure described in the caption.

4. I fully apologise for not seeing the authors’ response to Erica Moodie’s comments. I agree that they did respond.

 For the above reasons 1 and 2, I do not approve the paper as a whole. I do approve the methods and results, which provide useful insights. It is only the author’s interpretation of their results that I do not approve. An advantage of the open reviewing platform is that the paper remains available and those who disagree with my opinions remain able to read it.

 I am still learning how to review papers in non-conventional journals like this one. Usually I would not expect to review a 2 ^nd^ submission (such as this). I will not review a 3 ^rd^ submission.

I have read this submission. I believe that I have an appropriate level of expertise to state that I do not consider it to be of an acceptable scientific standard, for reasons outlined above.

# Response

**Published:** 2019-03-01

**Authors:** Jordi Cortés

**Affiliations:** Universitat Politècnica de Catalunya, Spain

**We are thankful for the comments from Prof. Ian White, which have helped us greatly in rethinking our paper.**

 1. We are debating whether “conventional clinical trials are designed to find differences with the implicit assumption that the effect is the same in all patients within the eligibility criteria”.

 The authors quote Paul Holland. The passage quoted is a careful definition of the “constant effect assumption”, but it does not support the authors’ argument - it does not claim that this assumption is required in randomised trials. Indeed elsewhere in his paper, Holland points out the value of an average causal effect:

 “The value of the average causal effect T is of potential interest for its own sake in certain types of studies. It would be of interest to a state education director who wanted to know what reading program would be the best to give to all of the first graders in his state. The average causal effect of the best program would be reflected in increases in statewide average reading scores.”

**We agree that Holland is using the concept of Average Causal Effect (ACE); however, he also mentions that " *The assumption of constant effect makes the value of the average causal effect relevant to every unit, and therefore, allows T to be used to draw causal inferences at the unit level *". However, we wonder to whom this quote is relevant: to health managers?; or to doctors giving specific advice to individual patients? In any case, our statement will still remain valid: health managers would like to know the amount of variability added by the treatment; or, even better, they would like to know any additional measure of the amount of deviations from the causal effect assumption.**

 I entirely agree with the authors that

 “Without this [constant effect] assumption, the value of the “average causal effect” is not enough to convey all the information about the treatment effect.”

 but I don’t agree that

 “If the effect randomly varies among the different units (as shown in panel D of Figure 1), we need to characterize its distribution: for example, by a normal distribution with its mean (delta) and standard deviation (SD).”

 My disagreement is over the word “need”. It would be really useful to characterize the distribution. But we usually can’t do that in RCTs (cross-over trials and n-of-1 trials being notable exceptions). If we can’t characterize the distribution, the mean is still useful, as in the reading program example above, or as in treatment of a group of patients.

**Thank you for this observation; however, we disagree. While it is certainly true that science currently cannot provide this information; that does not mean that we don’t *need* this information in order to provide individualized advice. For us, “need” is the correct word. Perhaps our disagreement is simply a question of nuance, for example, maybe Prof. White would prefer “need to be able to” or something similar.**

 As I said in my original review:

 “This is why the trials community worries so much about external generalisability: for example, if a trial treated 60% women and 40% men and showed a benefit of treatment, then a clinician treating women and men in the same ratio can be confident of giving a benefit overall, but a clinician treating women and men in a different ratio cannot be so confident.”

**Thank you, although we disagree. As stated before: maybe some patients may rely on an average treatment. However, the best clinicians would like to know the effect on both subgroups: this is why we “need” to know the “distribution” of the effect size among the population.**

 2. Here we are debating whether the authors’ conclusions follow validly from the observed reduction in variance in the treated arm. The authors have included plenty of caveats. But their primary statement (in the abstract) remains “treated patients … would not require further precision medicine”: I still disagree with this.

**Thank you, although we still disagree with Prof. Ian White’s interpretation.**

**If the constant effect assumption does not hold, new paradoxes may appear. For example, we agree that a different effect in some units may justify a tailored recommendation. However, in the case of a reduced variance with a similar mean (as in panel E, Figure 1), more patients would finish within reference or “normality” values, which may be interpreted as the goal of interventions.**

 Other over-statements in the discussion are

  “When both arms have equal variances, then the simplest interpretation is that the treatment effect is constant, thus rendering futile any search for predictors of differential response.” (p9)

**Again, thank you, although we disagree.** **As a constant ( *effect*) is independent of any variable, we wonder why this should be considered an “over-statement”**

 “For most trials, the variability of the response to treatment changes scarcely or even decreases, which suggests that precision medicine’s scope may be less than what is commonly assumed” (p10)

**As before.**

**“**There is evidence of effect variation in around 1 out of 7 trials, suggesting a limited role for tailored interventions” (p10)

**Again, “idem”: we also disagree with Prof. Ian White’s interpretation.**

 3. I find the presentation of the methods and results clearer now, though some parts remain poorly explained:

 In the formula for I^2, nu^2 is not the expected value of the error variance, since the latter is not a random variable.

**Thank you. We have corrected it: *“nu^2 is the mean of the error variances nu_i^2”***

 The formulae for V[log(V_OT/V_OC)] and V[log(V_OT/V_BT)] are in fact formulae for their within-study variances nu_i^2: this should be made clear. As written, they wrongly appear to be the total variances, which also involve tau^2

**Thank you. We have clarified this issue adding this sentence before the formulas.**

**“Thus, the within-study variance was estimated using the following formulas:”**

 In Table 1, “Random model” is an inadequate description of the complex procedure described in the caption.

**Thank you. As a consequence of your suggestions, we have decided to expand the table by specifying the 3 performed analyses based on: 1) the random effects model; 2) the heuristic method of eliminating one-to-one studies (what we previously called "random model" and now we call "heuristic method"); 3) the classic tests of variance comparison.**

 4. I fully apologise for not seeing the authors’ response to Erica Moodie’s comments. I agree that they did respond.

**Thank you.**

 For the above reasons 1 and 2, I do not approve the paper as a whole. I do approve the methods and results, which provide useful insights. It is only the author’s interpretation of their results that I do not approve. An advantage of the open reviewing platform is that the paper remains available and those who disagree with my opinions remain able to read it.

**Thank you. We are happy Prof White thinks it “provides useful insights” and that he likes that it should “remain available”. As we interpret his comments, he appears to believe that the paper is now a worthwhile contribution to science. Anyway, we wonder if a non-indexed paper could be considered as “available”**

 I am still learning how to review papers in non-conventional journals like this one. Usually I would not expect to review a 2nd submission (such as this). I will not review a 3rd submission.

 Competing Interests: No competing interests were disclosed. I have read this submission. I believe that I have an appropriate level of expertise to state that I do not consider it to be of an acceptable scientific standard, for reasons outlined above.

**It seems to us that we agree more than we disagree with Prof. Ian White.  We agree that the assumption of a constant effect is as unrealistic as some statements from the Normal Gauss-Laplace distribution. However, the constant effect has nevertheless served as a useful assumption for facilitating medical and social decisions arrived at on the basis of evidence-based medicine. The relevant question is not whether or not the assumption is true, but how much the effects deviate from a constant model. This is the point of our proposal, namely that it is necessary to quantify how much variability is added by the treatment.**

**In any case, we would like to thank again Prof. Ian White. Despite having different interpretations, he has helped us to significantly improve the article.**

# Peer review: Reviewer response for version 2

**DOI:** 10.5256/f1000research.16548.r34991

**Published:** 2018-07-27

**Authors:** Saskia le Cessie

**Affiliations:** 1 Department of Clinical Epidemiology, Leiden University Medical Center, Leiden, The Netherlands; 2 Section Medical Statistics, Department of Biomedical Data Science, Leiden University Medical Center, Leiden, The Netherlands

Although the paper has been improved, there are still important shortcomings in the abstract, introduction, description of the methods and the discussion.

1. I completely agree with Ian White. The statement in the abstract: “However, conventional clinical trials are designed to find differences with the implicit assumption that the effect is the same in all patients within the eligibility criteria”,  is definitely not true. Trials are designed to answer questions about **average** effects in populations: what would happen if everyone would be treated with treatment A versus treatment B. This does not imply that the effect in all subgroups or in all individuals is the same. This also apply to the discussion with statements like "Evidence-Based Medicine operates under the paradigm of a constant effect assumption"
2. The description of the methods is insufficient. The main fitted model has two random effect S_i and e_i, and the model is therefore not estimable. You do not clearly state that you circumvent this problem by estimating the variance  nu_i  from the sample sizes in the two trial arms.  
3. The parameter mu is not the average variance ratio, it is the logarithm of it. And the notation should be s_i ~N(0, tau^2) instead of s_i ~N(0, tau), idem e_i ~N(0, nu_i^2).
4. The definition of I^2 is not clear,  it is unclear what nu^2 is.
5. I am uncertain about the added value of using I^2. Why not just look at the size of tau, and use it to determine the prediction interval for the ratio of variances?
6. It is unclear how the model to assess homoscedasticity over time is formulated.
7. In the heading of Table 1 it is now explained how the distinction between increased variability, decreased variability and not changed is made. Please add this information to the methods section.
8. I can see how the distinction between increased, decreased and not changed stability based on the F-tests is made (although the term masked tests is unknown to me). However the random model method is rather heuristic. What is meant by a “neglectable heterogeneity”? And what is meant by” studies are removed one by one until achieving an estimated value of tau, similar to the reference model”. Which reference model? What difference is considered to be similar?
9. The presentation of the results is in some parts unclear. e.g. “The estimated baseline heterogeneity”. I assume that this is the estimate of the between study variance of an analysis with the log variance-ratio at baseline as dependent variable. Is that correct?  Page 6. “heterogeneity was almost doubled”. Do you mean that tau is doubled? Or tau^2? “The sensitive analysis” To which analysis do you refer here?
10. The word “outcome” has multiple meanings. Sometimes it is the outcome variable, sometimes it is outcome variable, measured at end of the study. See for example the legend of  Figure 3: the terms “variance of outcome” and “variance of outcome at baseline” are not very clear. Please reword.
11. In the discussion, you still equate heteroscedastic with precision medicine e.g. sentences like  “a rough estimate of the proportion of interventions with different variability that would require more precise medicine”.
12. Page 7 The part starting with "Considering the most" until "Provided there are no differences in means, the latter implies a larger proportion of “cured” patients, within the normality range.” I did not understand this part. Why do you assume that there are no differences in means? And what do you mean with normality range?
13. Page 9: “First, the heterogeneity found in our analysis indicates that the observed lower variability in the experimental arm cannot be extrapolated to all individual studies.” This remark is unclear.

I have read this submission. I believe that I have an appropriate level of expertise to state that I do not consider it to be of an acceptable scientific standard, for reasons outlined above.

# Response

**Published:** 2018-11-06

**Authors:** Jordi Cortés

**Affiliations:** Universitat Politècnica de Catalunya, Spain

**We are sincerely thankful for the critical review from Prof. Saskia le Cessie. Next, we will try to clarify the raised issues.**

Although the paper has been improved, there are still important shortcomings in the abstract, introduction, description of the methods and the discussion.

1. I completely agree with Ian White. The statement in the abstract: “However, conventional clinical trials are designed to find differences with the implicit assumption that the effect is the same in all patients within the eligibility criteria”,  is definitely not true. Trials are designed to answer questions about **average** effects in populations: what would happen if everyone would be treated with treatment A versus treatment B. This does not imply that the effect in all subgroups or in all individuals is the same. This also apply to the discussion with statements like "Evidence-Based Medicine operates under the paradigm of a constant effect assumption"

**As this question deals with the same issue posed by Professor Ian White, we use more or less the same answer:**

**Paul Holland [1], in his paper about statistics and causal inference, stated: “ *The assumption of constant effect is that the effect of t on every unit is the same […]. [It] makes the value of the average causal effect relevant to every unit.*”**

**Without this assumption, the value of the “average causal effect” is not enough to convey all the information about the treatment effect. If the effect randomly varies among the different units (as shown in panel D of Figure 1), we need to characterize its distribution: for example, by means of a normal distribution with its mean (delta) and standard deviation (SD). Depending on the value of this SD, we may consider applying the intervention to the full population or not. In the case of an interaction with measurable baseline characteristics (as shown in panel C of Figure 1), we need to specify the different delta values for each group.**

**Without specification of the further parameters required to characterize a non-constant effect, the reader cannot distinguish whether: (a) the authors were looking for a non-constant effect, but they erroneously omitted those further details required to specify this sophisticated effect; or (b) they were just assuming a constant effect (perhaps unconsciously). We agree with Prof. White that authors should be fully transparent about those assumptions; and with Grissom and Kim [2] in the sense that those further sophisticated situations may require a broader approach than just looking at the means.**

**[1] HOLLAND, P & PAUL, W. (1986). Statistics and Causal Inference. Journal of the American Statistical Association, 81(396), 945-960.**

**[2] GRISSOM, R. J. & KIM, J. J. (2005). Effect Sizes for Research: a Broad Practical Approach. Lawrence Erlbaum Associates, Mahwah, NJ**

2.The description of the methods is insufficient. The main fitted model has two random effect S_i and e_i, and the model is therefore not estimable. You do not clearly state that you circumvent this problem by estimating the variance  nu_i  from the sample sizes in the two trial arms.

**You are right. Although we have extensively explained it in sections VI and VII of the supplementary material, we have clarified it in a new version of the manuscript:**

**Before: “As there is only one available measure for each study, both sources of variability cannot be empirically differentiated: (i) within study or random or that one related to sample size; and (ii) heterogeneity. In order to isolate the second, the first was theoretically estimated using the Delta method –as explained in Sections V and VI in Supplementary File 1.”**

**After: “As there is only one available measure for each study, both sources of variability cannot be empirically differentiated: (i) within study or random variability; and (ii) heterogeneity. To isolate the second, the first was estimated theoretically using either the Delta method in the case of comparison between-arm or using some approximation in the case of comparison over time (see details in Sections VI and VII of Supplementary File 1):**

**V[log(V_OT/V_OC )]=2/(n_OT-2)+2/(n_OC-2)      (between arms)**

**V[log(V_OT/V_BT )]=** **4/(n-1) - 2·log [1+(2·Corr[Y_OT,Y_BT ]^2)/(n^2/(n-1))]      (over time)”**

3. The parameter mu is not the average variance ratio, it is the logarithm of it. And the notation should be s_i ~N(0, tau^2) instead of s_i ~N(0, tau), idem e_i ~N(0, nu_i^2).

**Thanks. We have corrected it.**

4. The definition of I^2 is not clear,  it is unclear what nu^2 is.

**Thank you for the suggestion. Regarding the definition of I^2, we have added a new reference [1] that contains a broader explanation of its meaning. Regarding nu^2, we have removed the second definition (“the expected value of the error variance”), since it had already been previously defined.**

**[1] Higgins JPT, Thompson SG, Deeks JJ, et al.: Measuring inconsistency in meta-analyses. BMJ. 2003;327(7414):557-560.**

****

5. I am uncertain about the added value of using I^2. Why not just look at the size of tau, and use it to determine the prediction interval for the ratio of variances?

**In fact, although we define the measure of I^2, our explanations always underlie the interpretation of tau.**

**The question about why we do not use the prediction intervals is very appropriate. This was our initial idea, but Carlisle's paper [1] made us think that there could be some methodological deficiencies in the random assignments that involve artificially different variances between arms. However, after making the decision, we found that the number of studies with statistically different variances using our "heuristic" method or using the prediction intervals was very similar. This can be checked by comparing the results shown in Table 1 with the points outside the triangle in Figure 3.**

**[1] Carlisle JB: Data fabrication and other reasons for non-random sampling in 5087 randomised, controlled trials in anaesthetic and general medical journals. Anaesthesia. 2017;72(8):944–952. 28580651 10.1111/anae.13938**

6. It is unclear how the model to assess homoscedasticity over time is formulated.

**The methodology is explained in Section IV of the supplementary file. As the methodology was relatively similar to the comparison between groups, we decided to omit the details in the statistical analysis section so as not to wear down the reader.**

7. In the heading of Table 1 it is now explained how the distinction between increased variability, decreased variability and not changed is made. Please add this information to the methods section.

**Thank you for your advice. We have introduced two modifications.**

**First, just to clarify, we have added an additional introductory paragraph in the statistical analysis section mentioning the 2 methods before explaining them:**

***To compare the variances, two different methods have been used: one based on a random-effects model and the other based on classical variance comparison tests.***

**Second, it is our opinion that doubts arise in regard to how the random-effects model determines which studies have significantly different variances (since we believe it is obvious with variance comparison tests), therefore we have added the following explanatory text in the statistical analysis section:**

***Specifically, studies with more extreme outcomes were removed one by one until achieving an estimated value of τ similar to the one obtained from the reference model. These deleted studies were considered to be those that had significantly different variances, because the experimental treatment either increased or decreased the variance.***

8. I can see how the distinction between increased, decreased and not changed stability based on the F-tests is made (although the term masked tests is unknown to me). However the random model method is rather heuristic. What is meant by a “neglectable heterogeneity”? And what is meant by” studies are removed one by one until achieving an estimated value of tau, similar to the reference model”. Which reference model? What difference is considered to be similar?

**The term "masked" refered to the fact that this analysis was defined without looking at the data previously. To clarify, we have changed "masked specified" to "pre-specified". We agree that the method which obtains the studies with significantly different variances is heuristic. However, it provides almost the same number of significant studies as when using the limits defined by the funnel-plots in Figure 3.**

**The reference model is the one in which the variances of the two groups are compared at the baseline: It is the first model of Table S4 of the supplementary file. To clarify, we have added an additional sentence in the legend of Table 1:**

**“Reference model – the one that compares the variances of the response at baseline”.**

**Also, we have slightly modified the explanation of this heuristic method to clear up doubts:**

**Before: “studies with more extreme outcomes were removed one by one until achieving an estimated value of tau similar to the one obtained from the reference model.”**

**After: “studies with larger discrepancies in variances were removed one by one until the estimated value of tau was as close as possible to that of the reference model.”**

9. The presentation of the results is in some parts unclear. e.g. “The estimated baseline heterogeneity”. I assume that this is the estimate of the between study variance of an analysis with the log variance-ratio at baseline as dependent variable. Is that correct?  Page 6. “heterogeneity was almost doubled”. Do you mean that tau is doubled? Or tau^2? “The sensitive analysis” To which analysis do you refer here?

**You are right in all the matters you mention.**

**Baseline heterogeneity refers to the model that uses the logarithm of the baseline variances ratio as response.**

**In the new version of the manuscript, we have clarified that when we say that “heterogeneity is approximately doubled”, we refer to the estimated tau.**

**Before: “Heterogeneity was approximately doubled.”**

**After: “Estimated heterogeneity (hat_tau) was approximately doubled.”**

**Also, instead of using the term "sensitivity analysis", which is not very specific, we now specifically mention what analysis we are referring to.**

**Before: “These figures were slightly higher in the sensitive analysis.”**

**After: “These figures were slightly higher in the analysis based on the classical variance comparison tests.”**

10. The word “outcome” has multiple meanings. Sometimes it is the outcome variable, sometimes it is outcome variable, measured at end of the study. See for example the legend of  Figure 3: the terms “variance of outcome” and “variance of outcome at baseline” are not very clear. Please reword.

**We have modified the legend of Figure 3.**

**Before: “V_BT: Variance of the Outcome at baseline in the Treated arm”.**

**After: “V_BT: Variance at Baseline in the Treated arm”.**

**In the rest of the document, we think that there is no ambiguity in the use of the term "Outcome", since it is always explicit when we refer to the baseline value.**

11. In the discussion, you still equate heteroscedastic with precision medicine e.g. sentences like  “a rough estimate of the proportion of interventions with different variability that would require more precise medicine”.

**It is not our intention in this statement to make an equivalence between heteroscedasticity and personalized medicine, i.e.:**

**Heteroscedasticity <-> Precision medicine**

**With the use of a conditional in the sentence, we want to highlight that the studies where personalized medicine has more room are those in which different variability has been observed and, therefore, they are those of which we are sure that the effect of the treatment is not constant:**

**Heteroscedasticity -> Precision medicine**

12. Page 7 The part starting with "Considering the most" until "Provided there are no differences in means, the latter implies a larger proportion of “cured” patients, within the normality range.” I did not understand this part. Why do you assume that there are no differences in means? And what do you mean with normality range?

**We wanted to emphasize that a treatment that is not effective because it does not modify the mean of the outcome could be beneficial by decreasing the variance, since this implies that there is a greater number of patients within the reference range (we have changed the terminology “normality range” to “reference range” in order to avoid ambiguities). We have changed the text in the following way:**

**Before: “Provided there are no differences in means, the latter implies a larger proportion of “cured” patients within the normality range.”**

**After: “Even if there are no differences in means, lower variance implies a larger proportion of patients within the reference range.”**

13. Page 9: “First, the heterogeneity found in our analysis indicates that the observed lower variability in the experimental arm cannot be extrapolated to all individual studies.” This remark is unclear.

**Thank you. We have tried clarified this sentence.**

**Before: “First, the heterogeneity found in our analysis indicates that the observed lower variability in the experimental arm cannot be extrapolated to all individual studies.”**

**After: “First, there are studies where the variability in the response is glaringly different, indicating the presence of a non-constant effect.”**

**We sincerely thank the referee for the time devoted to her comments, because despite not having considered the manuscript acceptable, she has helped us to considerably improve the manuscript.**

# Peer review: Reviewer response for version 2

**DOI:** 10.5256/f1000research.16548.r35299

**Published:** 2018-06-20

**Authors:** Ian R. White

**Affiliations:** 1 Medical Research Council Clinical Trials Unit, Institute of Clinical Trials and Methodology, University College London, London, UK

I appreciate the authors’ careful response to my review. They have made a number of changes which have improved the manuscript. However, a number of errors remain which leave me unable to approve the manuscript. The key errors are:

1. I still do not accept the authors’ argument (stated in the abstract) that “conventional clinical trials are designed to find differences with the implicit assumption that the effect is the same in all patients within the eligibility criteria”. Their response document does not make a convincing case. I think the authors see that standard procedures - the Neyman-Pearson lemma, a sample size calculation - use a single parameter, and infer that these procedures assume a common treatment effect. But this single parameter is the between-group difference of means, which equates to the **average** treatment effect. There is no assumption that the treatment effect is the same for all individuals.
2. The observed reduction in variance does **not** imply that “treated patients … would not require further precision medicine” (abstract). If for example there is a subgroup of less ill patients who derive no benefit, then this would explain the observed data, but excluding this subgroup would leave us in the setting of equal variances, which as already argued does not imply no role for precision medicine.
3. The reporting, though much improved, is still unclear in some ways. For example,
  1. what is the “baseline heterogeneity (τ2)” that is estimated as 0.31 (p6)?
  2. what does this mean: “both sources of variability cannot be empirically differentiated: (i) within study or random or that one related to sample size; and (ii) heterogeneity”?
  3. Table 1 remains unclear to  me.

I am also disappointed that the authors have not replied to Erica Moodie’s critical comments.

I have read this submission. I believe that I have an appropriate level of expertise to state that I do not consider it to be of an acceptable scientific standard, for reasons outlined above.

# Response

**Published:** 2018-07-26

**Authors:** Jordi Cortés

**Affiliations:** Universitat Politècnica de Catalunya, Spain

**We are honestly thankful for the critical review from Prof. Ian White, especially for highlighting the discrepancies between his and our interpretation regarding the average treatment effect (ATE) and the constant effect.**

**Nevertheless, we have the deep conviction that, by some honest mistake, Prof. White did not see our separate responses to each reviewer. Based on his comment that we did not answer Erica Moodie, which we had done as well as some other comments.**

**From here, we’ll answer specific issues of this report.**

I appreciate the authors’ careful response to my review. They have made a number of changes which have improved the manuscript. However, a number of errors remain which leave me unable to approve the manuscript. The key errors are:

1. I still do not accept the authors’ argument (stated in the abstract) that “conventional clinical trials are designed to find differences with the implicit assumption that the effect is the same in all patients within the eligibility criteria”. Their response document does not make a convincing case. I think the authors see that standard procedures - the Neyman-Pearson lemma, a sample size calculation - use a single parameter, and infer that these procedures assume a common treatment effect. But this single parameter is the between-group difference of means, which equates to the average treatment effect. There is no assumption that the treatment effect is the same for all individuals.

**Paul Holland [1], in his paper about statistics and causal inference, stated: “ *The assumption of constant effect is that the effect of t on every unit is the same (…). (It) makes the value of the average causal effect relevant to every unit.*”**

**Without this assumption, the value of the “average causal effect” is not enough to convey all the information about the treatment effect. If the effect randomly varies among the different units (as shown in panel D of Figure 1), we need to characterize its distribution: for example, by a normal distribution with its mean (delta) and standard deviation (SD). Depending on the value of this SD, we may consider applying the intervention to the full population or not. In the case of an interaction with measurable baseline characteristics (as shown in panel C of Figure 1), we need to specify the different delta values for each group.**

**Without specification of the further parameters required to characterize a non-constant effect, the reader cannot differentiate between: (a) the authors were looking for a non-constant effect, but they erroneously omitted those further details required to specify this sophisticated effect; or (b) they were just assuming a constant effect (perhaps subconsciously). We agree with Prof. White that authors should be fully transparent about those assumptions; and with Grissom and Kim [2] in the sense that those further sophisticated situations may require a broader approach than just looking at the means.**

**[1] HOLLAND, P & PAUL, W. (1986). Statistics and Causal Inference. Journal of the American Statistical Association, 81(396), 945-960.**

**[2] GRISSOM, R. J. & KIM, J. J. (2005). Effect Sizes for Research: a Broad Practical Approach. Lawrence Erlbaum Associates, Mahwah, NJ.**

2. The observed reduction in variance does not imply that “treated patients … would not require further precision medicine” (abstract). If for example there is a subgroup of less ill patients who derive no benefit, then this would explain the observed data, but excluding this subgroup would leave us in the setting of equal variances, which as already argued does not imply no role for precision medicine.

**This is the essential criticism. It was raised (and answered) in points 1 to 3 of the previous review from Prof White. We have added a new section to the supplementary file 1 ( *Conditions for homoscedasticity to hold without a constant effect under an additive model*) showing the conditions required for V[Y(1)]=V[Y(0)] under the additive model.**

**Essentially, if the effect was random, we need the correlation between the effect and V[Y(0)] to be exactly:**

- ½ * Sigma_effect/Sigma_Y(0).

**In the Discussion section of the 2 ^nd^ version of the paper, we did already include an extended explanation:**

**“Our second objective was (...) the remaining 80% of the studies agrees with the design assumption of a constant effect.”**

3. The reporting, though much improved, is still unclear in some ways. For example,

a) what is the “baseline heterogeneity (τ2)” that is estimated as 0.31 (p6)?

**This was previously raised by Prof. Ian White in the first revision (“Methods, point 2”), which offered us the opportunity to improve our presentation (see our previous answer).**

**It is an estimate of the variability of the logarithm of the ratio of variances at baseline. All the models are specified in the legend of Table S4 in Supplementary File 1.**

**Randomization should lead to this tau^2 being close to 0; and, in fact, by removing only the 4 most extreme studies reduces tau from 0.31 to 0.07, indicating that these 4 studies have some problems, such as in one study using outcomes that were obtained 1 month after the start of treatment as the baseline values [3].**

**[3] Hsieh LL, Kuo CH, Yen MF, et al.: A randomized controlled clinical trial for low back pain treated by acupressure and physical therapy. Prev Med. 2004; 39(1): 168–76**

b) what does this mean: “both sources of variability cannot be empirically differentiated: (i) within study or random or that one related to sample size; and (ii) heterogeneity”?

**The model can be defined without using scientific notation:**

**Response = Mean + Inter-study variability + Intra-study variability**

**By having a single measure per study, we do not have enough information to distinguish between intra-study variability and inter-study variability of the measure of interest. For this reason, the delta method was used to estimate the first and distinguish between them.**

c) Table 1 remains unclear to me.

**[See also our previous answer to Saskia Le Cessie comment 11.]**

**The white rows in Table 1 are the direct result obtained from a statistical test for comparing the variances between groups (F test) or before-after (paired test) in the experimental arms. From here, the studies are divided into not significant or significant in one or the other direction.**

**In the gray rows,  the significant studies are those that should be removed in order to obtain a heterogeneity as close as possible to the baseline (which supposedly should be zero by randomization). All those details were explained in either the text or the legend labels.**

I am also disappointed that the authors have not replied to Erica Moodie’s critical comments.

**Our reply to Erica Moodie was published on the F1000 website at the same time as the other two. We gave our best efforts in responding to her comments. We are grateful for her contribution even if we of course refuted any statements that we considered erroneous.**

# Peer review: Reviewer response for version 1

**DOI:** 10.5256/f1000research.14648.r31692

**Published:** 2018-04-03

**Authors:** Saskia le Cessie

**Affiliations:** 1 Department of Clinical Epidemiology, Leiden University Medical Center, Leiden, The Netherlands; 2 Section Medical Statistics, Department of Biomedical Data Science, Leiden University Medical Center, Leiden, The Netherlands

Review report. Does evidence support the high expectations placed in precision medicine? A bibliographic review. By J Cortés et al,

 Summary: a review of randomised trials with continuous outcomes, measured at baseline and at follow up has been conducted. The aim was to compare the variance of the outcome measure at baseline and follow-up and to compare the variances at follow-up between the treated and the control group. The authors argue that a difference in variances may indicate a heterogeneous treatment effect.

 General impression. The paper is well written and the results are of interest. The interpretation of the results is somewhat speculative but the authors discuss adequately the limitations.

 Remarks

1. Abstract, Background : “However, the conventional design of randomized trials assumes that each individual benefits by the same amount.” This is not a correct statement. In a randomised trial, the average treatment effect in the population is estimated, and no assumptions about homogeneity of treatment effects are made here. The authors probably mean that many researchers implicitly assume a homogeneous treatment effect when conducting a randomised trial, interpreting the  average treatment effect in the population as treatment effect at an individual level.
2. Introduction. I liked Figure 1 with the different explanations.
3. Methods and flow chart. The target population was parallel randomized clinical trials with quantitative/numerical outcomes. This is not true: trials with a survival time as outcome are also trials with a numerical (sometimes censored) outcome, but are not into the scope of your paper. So please mention that you are interested in trials with a numerical response variable which are measured both at baseline and at followup. In the Flow-chart, please check whether there were indeed 150 trials with a qualitative outcome, or whether there were 150 trials which did not satisfy the requirement of both having a baseline and a followup numerical measurement.
4. Statistical analysis. Here I got lost, the random mixed effects models should distinguish between random variability and heterogeneity, but how was unclear to me. Is adding the variance ratio at baseline needed to correct for the random variability? More details of the models and explanation of the different estimates of the model is needed, and should not only be given in the supplementary material.
5. Did you compare the Var(change) between the treated and control group? Power to detect differences here would be larger.
6. It may be of interest to perform a subgroup analysis in the studies where control is placebo
7. Supplementary material, section 4. The model has two random effects: s_i,   the heterogeneity between-study effect and e_i  the within sample error with variance nu^2 . I guess this should be nu_i, as each study has its own within sample error variance, estimated form the sample sizes in the two groups (as described in the material)?
8. The supplementary material did not clearly described which parameter(s) from the models reflected the heterogeneity. From the main text I derived that you used the mean effect mu to indicate the amount of heterogeneity. But then how to interpret the parameter tau?
9. Supplementary Table S4. Why not put this Table in Section 4, and make one overview of all the models fitted? And I guess that e_ij should be e_i here.
10. Results: I did not find Figure S1 and Figure S2 very informative. Why not just give a histogram of log(var_OT/var_CT) etc.
11. Table 1: How were the results from the random model obtained (the 11 increased, 19 decreased etc)?
12. Figure 3. Please explain what V_OT, V_OC etc is, as Figures should be self-explained.
13. I did not understand the second paragraph of the discussion. I guess that you want to say that the average treatment effect can be interpreted as an individual treatment effect, but I was confused at first by the words “non-observable patient treatment effect”.
14. Shocking to see that so many studies do not report measures of variability.
15. The fourth limitation: “the random effect model reveals additional heterogeneity”. To which result are you referring here, comparisons at baseline, followup or over time? The estimate of tau? Why should this be the result of methodological accuracies?
16. Figure G is of interest because this is a situation where precision medicine is of interest: for some patients treatment T would be a better choice, for others treatment C and by performing precision medicine the subgroups with different responses could be detected and tailored prescriptions could be given. This indicates that observed homoscedasticity in a study should be interpreted with care and background knowledge of a study is needed to assess whether a situation as in Figure 4 is plausible.

I have read this submission. I believe that I have an appropriate level of expertise to confirm that it is of an acceptable scientific standard, however I have significant reservations, as outlined above.

# Response

**Published:** 2018-06-03

**Authors:** Jordi Cortés

**Affiliations:** Universitat Politècnica de Catalunya, Spain

**JOINT ANSWER to Ian White and Saskia le Cessie**

**This is a general response to Ian White and Saskia Le Cessie on why we stated that the standard clinical trial design and analysis assume a constant effect.**

**In the following, (1) we update the standard sample size rationale; and (2) we explain why inflated variances may require precision medicine in just two general cases: (a) interaction, as represented in Fig. 1, panel C; and (b) random treatment effect, Fig. 1, panel D.**

1. **Under the Neyman-Pearson framework to determine sample size, a single effect size value Δ is specified under the alternative hypothesis H1, assuming in that way a constant effect, as in Fig. 1, panels A (H0) and B (H1).**
2. **We devise two situations that, because they result in higher variance, they would need personalized medicine:**
  - **Interaction between treatment and a baseline variable such us, for example, gender (Fig. 1, panel C). In this scenario there are two subpopulations (e.g., men and women) with different treatment effects that require the effect to be made further “precise”.**
  - **Random treatment effect on each patient (Fig. 1, panel D). In this scenario, the effect size does not depend on a known patient baseline characteristic and the only way to estimate the individual patient effect is by means of individualized trials (“n of 1” trials). **

 

**Those 2 hypothetical scenarios, lead to an increased variance. Conversely, scenarios E and F represent two similar situations (interaction and random effect) but result in reduced variance –without relevant changes on the average. Although we agree that in those two last scenarios leading to reduced variability the specific patient treatment effect may still be unknown because the outcome has reduced variability with a similar central overall position, we argue that patients in those situations were subject to “further control” (having more stable values within the boundaries of “normality”).**

**So, the usual sample size rationale specified by statisticians in trials assumes a constant, unique effect that agrees with the clinical and legal interpretation that the effect is the same – or at least similar enough to be considered homogeneous – for all the patients fulfilling the eligibility criteria.**

**To illustrate this secondary “argument”, we reviewed the sample size rationale for the last (at that time) 10 protocols published in Trials, and we found that all of them defined a single effect size (100%, two-sided 95% confidence interval from 69% to 100%). In addition, we have included a new column in Table S1 with the main analysis showing that the SAP in all those cases (10 out of 10, 95%CI from 69 to 100%) was also designed to estimate a single, constant effect.**

**We have modified Fig. 1 (panels E and F) to show decreasing variance treatment effects, but now without affecting the average. We have also improved the 2 following sentences:**

**Before [Abstract]:** **However, the conventional design of randomized trials assumes that each individual benefits by the same amount.**

**After [Abstract]: However, conventional clinical trials are designed to find differences with the implicit assumption that the effect is the same in all patients within the eligibility criteria.**

**Before [Introduction]:** **The assumption that the average effect equals the single unit effect underlies the rationale behind the usual sample size calculation, where only a single effect is specified. As an example, the 10 clinical trials published in the Trials Journal in October 2017 (see Supplementary material: Table S1) were designed under this scenario of a fixed, constant or unique effect in the sample size calculation.**

**After [Introduction]: The assumption of homoscedasticity in the usual calculations of sample size is better interpreted under the constant effect model (Figure 1, panels A, H _0_; and B, H _1_). As an example, the 10 clinical trials published in the Trials Journal in October 2017 (Table S1 of Supplementary material) were designed with only a constant for the effect size. Furthermore, all their analyses were designed to test (and estimate) a single constant for the effect size. In other words, there was mention of neither any possible interaction with baseline variables (Figure 1, scenarios C and E), nor of any random variability for the treatment effect (Figure 1, scenarios D and F); and thus, all those trials were designed to test a constant effect.**

**We have also updated the legend of Figure 1 to highlight that now panels C to F show only possible individual treatment effects on variances but not on means.**

**We are deeply grateful to Ian White and Saskia le Cessie for highlighting the need to clarify this crucial issue.**

**Saskia le Cessie**

**From here, we’ll answer specific issues**

Summary: a review of randomised trials with continuous outcomes, measured at baseline and at follow up has been conducted. The aim was to compare the variance of the outcome measure at baseline and follow-up and to compare the variances at follow-up between the treated and the control group. The authors argue that a difference in variances may indicate a heterogeneous treatment effect.

General impression. The paper is well written and the results are of interest. The interpretation of the results is somewhat speculative but the authors discuss adequately the limitations.

Remarks

**We are grateful to Prof. Saskia le Cessie for her suggestions, which definitively help us to improve our manuscript.**

1. Abstract, Background : “However, the conventional design of randomized trials assumes that each individual benefits by the same amount.” This is not a correct statement. In a randomised trial, the average treatment effect in the population is estimated, and no assumptions about homogeneity of treatment effects are made here. The authors probably mean that many researchers implicitly assume a homogeneous treatment effect when conducting a randomised trial, interpreting the average treatment effect in the population as treatment effect at an individual level.

**Yes, our impression is that at least some trialists are not aware of these assumptions. But the fact that we wanted to highlight is that trials are usually designed to provide evidence for just one parameter (in our context the “effect size” collected by the difference of means) without further specification, neither in the sample size rationale nor in the analysis of the further parameters required by precision medicine. We have addressed this point in the joint answer above.**

2. Introduction. I liked Figure 1 with the different explanations.

**Thank you. Please note that we have now updated panels C to F to isolate changes just in variance.**

3. Methods and flow chart. The target population was parallel randomized clinical trials with quantitative/numerical outcomes. This is not true: trials with a survival time as outcome are also trials with a numerical (sometimes censored) outcome, but are not into the scope of your paper. So please mention that you are interested in trials with a numerical response variable which are measured both at baseline and at follow-up. In the Flow-chart, please check whether there were indeed 150 trials with a qualitative outcome, or whether there were 150 trials which did not satisfy the requirement of both having a baseline and a follow-up numerical measurement.

**Thanks. We fully agree that discussion was introduced too late, and we have further clarified it in the Methods section and in the flow chart. The modifications are described below.**

***Before [Methods]:*** ***Our target population was parallel randomized clinical trials with quantitative outcomes***

**After [Methods]:** **Our target population was parallel randomized clinical trials with quantitative outcomes (not including time-to-event studies)**

***Before [Flow chart]:*** ***Qualitative outcome***

**After [Flow chart]:** **Categorical or time-to-event outcome**

4. Statistical analysis. Here I got lost, the random mixed effects models should distinguish between random variability and heterogeneity, but how was unclear to me. Is adding the variance ratio at baseline needed to correct for the random variability? More details of the models and explanation of the different estimates of the model is needed, and should not only be given in the supplementary material.

**Thanks.** **The model includes the (logarithm of the) baseline variances ratio because some imbalances in the initial variability between groups (after randomization) can occur simply by chance. It is foreseeable that these baseline differences in variability may influence the final differences in variability. This baseline log-ratio was highly significant (p < 0.0001)** **in the model.**

**All your suggestions related to the statistical analysis (4, 7, 8, 9 and 11) and the random effects model have been addressed through a clearer and longer explanation of the model in the statistical analysis section (detailed** **here** **and in the manuscript)**

**Nevertheless, we provide the following rule of thumb for interpreting the parameters μ (heteroscedasticity) and I ^2^ (heterogeneity) of the random-effects model.**

**mu < 0 -->     On average, studies have lower variability in the experimental arm.**

**mu > 0 -->     On average, studies have greater variability in the experimental arm.**

**I^2 < 25% --> As the point estimate of heterogeneity is not high enough,** **mu** **is constant throughout all the studies.**

**I^2 < 25% --> As the point estimate of heterogeneity is high,** **mu** **does not apply to every single study.**

**mu < 0 & I^2 < 25% -->  Not one study requires precision medicine.**

**mu < 0 & I^2 > 25% -->  Some studies require precision medicine.**

**mu > 0 & I^2 < 25% -->  All studies require precision medicine.**

**mu > 0 & I^2 > 25% -->  Most studies require precision medicine.**

**[The threshold of 25% for I^2 is based on PRISMA Statement [1] that considers values under this cutpoint as low.]**

**The estimates of these parameters in our data were mu = -0.12 and I^2 = 80.8%, which implies that** **some studies require precision medicine.**

1. ***Liberati A, Altman DG, Tetzlaff J, Mulrow C, Gøtzsche P, et al. (2009) The PRISMA statement for reporting systematic reviews and meta-analyses of studies that evaluate health care interventions: Explanation and elaboration. PLoS Med 6: e1000100.***

5. Did you compare the Var(change) between the treated and control group? Power to detect differences here would be larger.

**Strongly agree. For high correlations between baseline and outcome, it follows that V (log (V _Ox_/V _Bx_)) < V (log (V _Ox_)), as can be seen in Appendix VII of the supplementary material. However, just 95 out of 208 studies provide the Var(change) or the baseline-final correlation that would allow this analysis.**

6. It may be of interest to perform a subgroup analysis in the studies where control is placebo

**In fact, we performed this subgroup analysis beforehand without obtaining relevant results. We decided not to include or mention it because the distinction between a treatment called "placebo" and an "active" treatment is not clear. “Placebo” is defined as a simulator of the experimental treatment that tries to emulate its characteristics; but in some studies “control” may equal “best medical treatment”, which is also provided to “treated” patients, such that “Placebo” is complemented by the standard intervention. Because of this ambiguity in the classification, we decided to omit this information. As illustrative examples, we mention the following included studies:**

**- *Ghaleiha A, Mohammadi E, Mohammadi M, et al. Riluzole as an adjunctive therapy to risperidone for the treatment of irritability in children with autistic disorder: a double­blind, placebo­controlled, randomized trial. Paediatr Drugs 2013; 15:505–514. *The patients in the reference group took placebo in addition to risperidone (titrated up to 2 or 3 mg/day based on bodyweight) for 10 weeks.**

**- *Carroll MW, Jeon D, Mountz JM, et al. Efficacy and safety of metronidazole for pulmonary multidrug-resistant tuberculosis. Antimicrob Agents Chemother 2013; 57:3903-9*. The patients in the reference group took placebo for 8 weeks in addition to an individualized background regimen.**

7. Supplementary material, section 4. The model has two random effects: s_i,   the heterogeneity between-study effect and e_i  the within sample error with variance nu^2 . I guess this should be nu_i, as each study has its own within sample error variance, estimated form the sample sizes in the two groups (as described in the material)?

**Thank you. We have corrected the typo including the subscript both in the Methods section and in the supplementary material: “nu_i”**

8. The supplementary material did not clearly describe which parameter(s) from the models reflected the heterogeneity. From the main text I derived that you used the mean effect mu to indicate the amount of heterogeneity. But then how to interpret the parameter tau?

**Thanks. Tau reflects the heterogeneity in the assessment of the heteroscedasticity throughout the studies. Following this suggestion and similar comment of Professor Ian White, we have tried to clarify that mu is a measure of heteroscedasticity and tau is a measure of the heterogeneity of the former throughout all the studies. See also the answer to question 4 for more clarification.**

9. Supplementary Table S4. Why not put this Table in Section 4, and make one overview of all the models fitted? And I guess that e_ij should be e_i here.

**Thank you, we have corrected the subscript typo: “e_i”**

**And yes, your suggestion facilitates readability. We have interspersed all the tables and figures of the supplementary material in their respective sections.**

10. Results: I did not find Figure S1 and Figure S2 very informative. Why not just give a histogram of log(var_OT/var_CT) etc.

**We have kept Figures S1 and S2 because we believe that they provide additional information about whether or not the increase (or decrease) in the variability in the outcome of the experimental arm depends on the outcome variability of the control arm (or on the baseline variability of the experimental group). However, we have also added the histograms you mention in order to summarize the essential information. The histograms can be seen** **here** **or in the Supplementary material.**

11. Table 1: How were the results from the random model obtained (the 11 increased, 19 decreased etc)?

**We have obtained them as the studies that had to be removed in order to obtain heterogeneity (i.e., tau) similar to the baseline (which we expect to be null by randomization). We have tried to clarify this point in the legend of the table:**

**“…or (2) number of studies that have to be deleted from the random-effects model in order to achieve a negligible heterogeneity (studies with more extreme outcome were removed one by one until achieving an estimated value of τ similar to the one obtained from the reference model. See Methods section for more details…)”**

12. Figure 3. Please explain what V_OT, V_OC etc is, as Figures should be self-explained.

**Thanks. We have included a legend in this figure explaining these abbreviations:**

***V_OT: Variance of the Outcome in the Treated arm***

***V_OC: Variance of the Outcome in the Control arm***

***V_BT: Variance of the Outcome at baseline in the Treated arm***

13. I did not understand the second paragraph of the discussion. I guess that you want to say that the average treatment effect can be interpreted as an individual treatment effect, but I was confused at first by the words “non-observable patient treatment effect”.

**You are right. We say “non-observable” for the fundamental problem of causal inference (both potential responses are not observable in the same patient), which avoids seeing the treatment effect at the individual level. We have clarified this point:**

**Before:** **This means that treatment effects obtained by comparing the means between groups can be used to estimate both the averaged treatment effect and the non-observable patient treatment effect.**

**After: This means that the average treatment effect can be interpreted as an individual treatment effect (not directly observable).**

14. Shocking to see that so many studies do not report measures of variability.

**Yes. It is really surprising that 61.6% of studies do not report the variability either at baseline or at the end of the study. Although CONSORT advises it, this guideline does not provide the historical data on this practice with which it can be compared.**

15. The fourth limitation: “the random effect model reveals additional heterogeneity”. To which result are you referring here, comparisons at baseline, followup or over time? The estimate of tau? Why should this be the result of methodological accuracies?

**We are referring to the main analysis: comparison between arms. Nevertheless, this sentence could be applied to all analyses. Heterogeneity among studies is measured by tau (see response to question 4).**

**We stated that methodological inaccuracies can be derived in the presence of heterogeneity. In an ideal scenario of constant treatment effect in all the studies, the only thing that could lead to heterogeneity in the model would be methodological inaccuracies such as those mentioned in the manuscript or in the referenced paper of Carlisle: transcription errors, insufficient follow-up time for being able to observe this constant effect, or the manipulation of the results in order to achieve greater impact.**

16. Figure G is of interest because this is a situation where precision medicine is of interest: for some patients treatment T would be a better choice, for others treatment C and by performing precision medicine the subgroups with different responses could be detected and tailored prescriptions could be given. This indicates that observed homoscedasticity in a study should be interpreted with care and background knowledge of a study is needed to assess whether a situation as in Figure 4 is plausible.

**Fully agree, although this is a highly sophisticated scenario that we hope will not be viewed as a frequent scenario.**

**Of course, we think that personalized medicine has already been demonstrated to be effective in some areas. Our point is that unless those demonstrations exist, most interventions should be routinely administrated to all patients fulfilling eligibility criteria.**

# Peer review: Reviewer response for version 1

**DOI:** 10.5256/f1000research.14648.r31694

**Published:** 2018-03-23

**Authors:** Erica E.M. Moodie

**Affiliations:** 1 Department of Epidemiology, Biostatistics and Occupational Health, McGill University, Montreal, QC, Canada

The authors have performed a review of a sample of clinical trials conducted every three years from 2004-2013 to examine whether there exists post-treatment heterogeneity in participants responses with premise that lack of heterogeneity suggests that precision medicine is not warranted.

 While the question is one that should be asked. However, the study carried out is not suited to answering the question as it has been conducted in randomized trials where there is typically little heterogeneity. That is, the authors have performed a perfectly reasonable analysis that cannot answer the pertinent question. It is well known that randomized trials tend to be populated by homogenous population (more white, more male, etc.) – see, for example Oh et al. (2015) Diversity in Clinical and Biomedical Research: A Promise Yet to Be Fulfilled. PLoS Med 12(12): e1001918, Caplan & Friesen P (2017) Health disparities and clinical trial recruitment: Is there a duty to tweet? PLoS Biol 15(3): e2002040 and the references therein – or indeed many other papers on this topic. This may be in part a function of recruitment strategies and also by design, as trialists (particularly those testing new therapies) often determine inclusion criteria to target the (potentially homogeneous) segment of the population who might show the greatest response to the treatment. Thus, the authors have chosen to study a population that is likely to be homogeneous and not reflective of real-world clinical care. There are numerous examples covariate-tailored treatment algorithms, from the choice of hormonal therapies for women diagnosed with estrogen-receptor-positive, HER2-negative breast cancer to the choice of ACE inhibitors vs. calcium channel blockers for hypertension, that the authors choose to overlook as cases where we have learned about previous patients *with particular characteristics* to learn about future similar patients.

I have read this submission. I believe that I have an appropriate level of expertise to state that I do not consider it to be of an acceptable scientific standard, for reasons outlined above.

# Response

**Published:** 2018-06-03

**Authors:** Jordi Cortés

**Affiliations:** Universitat Politècnica de Catalunya, Spain

The referee’s objections can be summarized in two points:

(1) “There are numerous examples of covariate-tailored treatment algorithms.”

(2) “Randomized trials tend to be populated by homogenous populations”, which in turn does not reflect a real population’s existing variability.

We understand the reviewer’s comments, but we disagree with the reviewer’s conclusions:

(1) Our work does not intend to completely invalidate precision medicine. We are not stating that there are not “examples of covariate-tailored treatments”; rather that (Abstract): “We found that the outcome variance was more often smaller in the intervention group, suggesting that treated patients may end up pertaining more often to reference or normality values and thus would not require further precision medicine”. This was already stated in the discussion: “these findings do not invalidate precision medicine in all settings.” Thus in the quite wide settings of our trials, we found little evidence that precision medicine would be of any use.

(2) The referee argues that trials have “too many” selection criteria to reflect “a real population”. This is a standard criticism of explanatory clinical trials, suggesting that the selection criteria are usually “too many”. And we agree, because our point is that most trials have “enough” selection criteria to provide a homogeneous effect. Furthermore, we also agree that we can only talk about “published trials with eligibility criteria”. As for whether those selection criteria should be used to define the target population in clinical guidelines, there is no further need to tailor precision medicine.

Dr. Moodie argues that our results do not answer the question that is posed. We also disagree. The issue of heterogeneity is obviously one that bedevils the generalizability of clinical trials. However, these are randomized comparisons; so, in the absence of a treatment effect we would expect the two arms to be comparable, no matter how heterogeneous the underlying population. The fact that even in the presence of a treatment effect there was little evidence of heterogeneity suggests there will be little scope for precision medicine in these populations. One might argue that with a more heterogeneous population, there is more scope to detect the few non-responders who would not form part of a general trial population. This does not invalidate our results; rather it argues for much larger trials with a more heterogeneous population. The point is that in the absence of these the evidence base for precision medicine is weak.

# Peer review: Reviewer response for version 1

**DOI:** 10.5256/f1000research.14648.r30604

**Published:** 2018-03-02

**Authors:** Ian R. White

**Affiliations:** 1 Medical Research Council Clinical Trials Unit, Institute of Clinical Trials and Methodology, University College London, London, UK

This paper considers randomised trials (RCTs) of treatment versus control with a quantitative outcome. It observes that *if* treatment effects are homogeneous (the same for all trial participants) *then* the outcome variance will be the same in both trial arms. It therefore reviews the extent to which the outcome variance is the same across trial arms in 208 published RCTs. It finds 41 RCTs with significant differences in outcome variance, and that it is more common for the outcome variance to be smaller in the treatment arm rather than larger.

 My overall comment is that the analysis results are useful, but they need to be made clearer, and the interpretation should be much more cautious. Points marked * must be addressed to make the article scientifically sound (with ** the top priority).

**Background**

*Abstract, background: “The conventional design of randomized trials assumes that each individual benefits by the same amount.” This is also asserted elsewhere in the paper, but it is not true. From a causal inference perspective, a RCT estimates the average causal effect, which is well defined in the presence of treatment effect heterogeneity. This is why the trials community worries so much about external generalisability: for example, if a trial treated 60% women and 40% men and showed a benefit of treatment, then a clinician treating women and men in the same ratio can be confident of giving a benefit overall, but a clinician treating women and men in a different ratio cannot be so confident. This point (repeated elsewhere) is not essential to the paper’s argument, so should be removed.
*Similarly, the argument “The assumption that the average effect equals the single unit effect underlies the rationale behind the usual sample size calculation, where only a single effect is specified” (Introduction) is false. Sample size calculations relate only to comparisons of group averages.

**Methods**

 The methods used appear entirely appropriate. However they are not well described.

*Terminology must be improved. For example, the key outcome in this study is the ratio of variances between treatment and control arms, and this (or its opposite) is variously called “homoscedasticity”, “heterogeneity”, even “concordance”. The authors should choose a term and stick with it. Similarly for the “random mixed effects model” which later becomes the “random model”. (I’m going to use “homoscedasticity” and “random-effects model”.)
2. The authors are doing a meta-analysis, even though they don’t call it that, so the term “heterogeneity” should be reserved for “variation between studies”, i.e. tau^2 in the random effects models.
*It’s not clear to me what the “random model” results in Table 1 are. Since this is a model across studies, how can it count individual studies? If empirical Bayes estimates of study-specific effects are being tested, this must be explained.
4. Trials that are “significant” are combined - “Subgroup analyses suggest that only significant interventions had an effect on reducing variability” - but interventions that increase the mean should be separated from those that decrease the mean. The later conclusion that “The variability seems to decrease for treatments that perform significantly better than the reference” suggests a different distinction (better/worse is not the same as larger/smaller because outcomes may be positive or negative) and is not supported by the results presented.
5. Abstract, Results: “The adjusted point estimate of the mean ratio (treated to control group) of the outcome variances” is not clear without reading the whole text. Again, defining a term (“outcome variance ratio”?) will help.
*Table 1, “variability is… increased”: from the text, this means “significantly increased”, which should be clarified.

**Interpretation**

 The results may be interpreted in many ways, which are sensibly discussed by the authors.

 Most importantly, treatment effect homogeneity implies homoscedasticity, but the converse (“homoscedasticity implies treatment effect homogeneity”) is not true: this is demonstrated very nicely in Figure 4.

 Homoscedasticity is scale-dependent: for example, it may be removed (or created) by a log transformation (mentioned in the Discussion).

*The authors omit one alternative explanation of homoscedasticity over time: clinical trial populations have eligibility criteria at baseline which may limit baseline variance. For example, a hypertension trial might recruit patients with baseline SBP between 140 and 159 mm Hg. In this case, variance is very likely to naturally increase over time.
**The authors’ conclusions ignore the alternative interpretations noted above. Here are some examples which are illogical:
  - Abstract, Conclusions: “the variance was more often smaller in the intervention group, suggesting, if anything, a reduced role for precision medicine”, and Discussion: “variability tends to be reduced on average after treatment, thus making precision medicine dispensable in most cases”. This is actually false. If a study finds smaller variance in the treated group then we DO have evidence of treatment effect heterogeneity, and indeed the treatment may be doing exactly what medicine should do - making the sickest better while not harming the less sick.
  - Introduction: “If this homoscedasticity holds, there is no need to repeat the clinical trial once a new possible effect modifier becomes measurable” - again, this wrongly assumes the converse stated above.
  - Discussion: “When both arms have equal variances, then an obvious default explanation is that the treatment is equally effective for all, thus rendering the search for predictors of differential response futile”: this is illogical.
  - Discussion: “For most trials, subjects vary little in their response to treatment, which suggests that precision medicine’s scope may be less than what is commonly assumed.” : this is also illogical.
*In the light of the above arguments, I find the statement (Abstract, Conclusions) that “Homoscedasticity is a useful tool for assessing whether or not the premise of constant effect is reasonable” to be highly debatable. Logic suggests it gives a lower bound on the extent of usefulness of precision medicine, and the results of this study do not add any more to this.
*The objectives in the Discussion should be the same as those stated in the Introduction.

**Source data**

1. I had trouble opening the source data both in Excel (since the csv file is in fact semi-colon-delimited) and in Stata (which was thrown by line 80). Could it be provided in a more convenient format or with some notes?

I have read this submission. I believe that I have an appropriate level of expertise to confirm that it is of an acceptable scientific standard, however I have significant reservations, as outlined above.

# Response

**Published:** 2018-06-03

**Authors:** Jordi Cortés

**Affiliations:** Universitat Politècnica de Catalunya, Spain

**JOINT ANSWER to Ian White and Saskia le Cessie**

**This is a general response to Ian White and Saskia Le Cessie on why we stated that the standard clinical trial design and analysis assume a constant effect.**

**In the following, (1) we update the standard sample size rationale; and (2) we explain why inflated variances may require precision medicine in just two general cases: (a) interaction, as represented in Fig. 1, panel C; and (b) random treatment effect, Fig. 1, panel D.**

1. **Under the Neyman-Pearson framework to determine sample size, a single effect size value Δ is specified under the alternative hypothesis H1, assuming in that way a constant effect, as in Fig. 1, panels A (H0) and B (H1).**
2. **We devise two situations that, because they result in higher variance, they would need personalized medicine:**
  - **Interaction between treatment and a baseline variable such us, for example, gender (Fig. 1, panel C). In this scenario there are two subpopulations (e.g., men and women) with different treatment effects that require the effect to be made further “precise”.**
  - **Random treatment effect on each patient (Fig. 1, panel D). In this scenario, the effect size does not depend on a known patient baseline characteristic and the only way to estimate the individual patient effect is by means of individualized trials (“n of 1” trials). **

 

**Those 2 hypothetical scenarios, lead to an increased variance. Conversely, scenarios E and F represent two similar situations (interaction and random effect) but result in reduced variance –without relevant changes on the average. Although we agree that in those two last scenarios leading to reduced variability the specific patient treatment effect may still be unknown because the outcome has reduced variability with a similar central overall position, we argue that patients in those situations were subject to “further control” (having more stable values within the boundaries of “normality”).**

**So, the usual sample size rationale specified by statisticians in trials assumes a constant, unique effect that agrees with the clinical and legal interpretation that the effect is the same – or at least similar enough to be considered homogeneous – for all the patients fulfilling the eligibility criteria.**

**To illustrate this secondary “argument”, we reviewed the sample size rationale for the last (at that time) 10 protocols published in Trials, and we found that all of them defined a single effect size (100%, two-sided 95% confidence interval from 69% to 100%). In addition, we have included a new column in Table S1 with the main analysis showing that the SAP in all those cases (10 out of 10, 95%CI from 69 to 100%) was also designed to estimate a single, constant effect.**

**We have modified Fig. 1 (panels E and F) to show decreasing variance treatment effects, but now without affecting the average. We have also improved the 2 following sentences:**

**Before [Abstract]:** **However, the conventional design of randomized trials assumes that each individual benefits by the same amount.**

**After [Abstract]: However, conventional clinical trials are designed to find differences with the implicit assumption that the effect is the same in all patients within the eligibility criteria.**

**Before [Introduction]:** **The assumption that the average effect equals the single unit effect underlies the rationale behind the usual sample size calculation, where only a single effect is specified. As an example, the 10 clinical trials published in the Trials Journal in October 2017 (see Supplementary material: Table S1) were designed under this scenario of a fixed, constant or unique effect in the sample size calculation.**

**After [Introduction]: The assumption of homoscedasticity in the usual calculations of sample size is better interpreted under the constant effect model (Figure 1, panels A, H _0_; and B, H _1_). As an example, the 10 clinical trials published in the Trials Journal in October 2017 (Table S1 of Supplementary material) were designed with only a constant for the effect size. Furthermore, all their analyses were designed to test (and estimate) a single constant for the effect size. In other words, there was mention of neither any possible interaction with baseline variables (Figure 1, scenarios C and E), nor of any random variability for the treatment effect (Figure 1, scenarios D and F); and thus, all those trials were designed to test a constant effect.**

**We have also updated the legend of Figure 1 to highlight that now panels C to F show only possible individual treatment effects on variances but not on means.**

**We are deeply grateful to Ian White and Saskia le Cessie for highlighting the need to clarify this crucial issue.**

**Ian White**

**From here, we’ll answer specific issues**

This paper considers randomised trials (RCTs) of treatment versus control with a quantitative outcome. It observes that *if* treatment effects are homogeneous (the same for all trial participants) *then* the outcome variance will be the same in both trial arms. It therefore reviews the extent to which the outcome variance is the same across trial arms in 208 published RCTs. It finds 41 RCTs with significant differences in outcome variance, and that it is more common for the outcome variance to be smaller in the treatment arm rather than larger.

My overall comment is that the analysis results are useful, but they need to be made clearer, and the interpretation should be much more cautious. Points marked * must be addressed to make the article scientifically sound (with ** the top priority).

**We are grateful to Prof. Ian White for his suggestions, which will definitively help us to improve our manuscript.**

**Background**

1.*Abstract, background: “The conventional design of randomized trials assumes that each individual benefits by the same amount.” This is also asserted elsewhere in the paper, but it is not true. From a causal inference perspective, a RCT estimates the average causal effect, which is well defined in the presence of treatment effect heterogeneity. This is why the trials community worries so much about external generalisability: for example, if a trial treated 60% women and 40% men and showed a benefit of treatment, then a clinician treating women and men in the same ratio can be confident of giving a benefit overall, but a clinician treating women and men in a different ratio cannot be so confident. This point (repeated elsewhere) is not essential to the paper’s argument, so should be removed.

2. *Similarly, the argument “The assumption that the average effect equals the single unit effect underlies the rationale behind the usual sample size calculation, where only a single effect is specified” (Introduction) is false. Sample size calculations relate only to comparisons of group averages.

**Thanks again for highlighting this hugely important issue. We have addressed these two comments in the previous common answer.**

**Methods**

The methods used appear entirely appropriate. However they are not well described.

1. *Terminology must be improved. For example, the key outcome in this study is the ratio of variances between treatment and control arms, and this (or its opposite) is variously called “homoscedasticity”, “heterogeneity”, even “concordance”. The authors should choose a term and stick with it. Similarly for the “random mixed effects model” which later becomes the “random model”. (I’m going to use “homoscedasticity” and “random-effects model”.)

**Thanks. To simplify the notation, we have deleted the term “concordance”. We also reserved the term heterogeneity for the tau^2 statistic resulting from the mixed-effects model (see next answer). Furthermore, we have homogenized the terms for referring to the** **“random-effects model” throughout the text.**

2. The authors are doing a meta-analysis, even though they don’t call it that, so the term “heterogeneity” should be reserved for “variation between studies”, i.e. tau^2 in the random effects models.

**We appreciate this insightful observation. In the random-effects model, we measured heteroscedasticity with the mu parameter, and heterogeneity between studies, through tau^2.** **In order to clarify this as much as possible, we have specified in the Methods section that tau^2 is used for measuring heterogeneity; and this has also been included between brackets in the Results section:**

**“The estimated value of tau^2 provides a measure of heterogeneity, that is, to what extent the value of mu is applicable to all studies. The larger tau^2 is, the less the homogeneity”**

3. *It’s not clear to me what the “random model” results in Table 1 are. Since this is a model across studies, how can it count individual studies? If empirical Bayes estimates of study-specific effects are being tested, this must be explained.

**We used the Delta method to estimate the within study variability (specifically, the variance of the logarithm of the outcome variance ratio). We have included this explanation in the Methods section: “As there is only one available measure for each study, both sources of variability cannot be empirically differentiated: (i) within study or random or that one related to sample size; and (ii) heterogeneity. In order to isolate the second, the first was theoretically estimated using the Delta method –as explained in Sections V and VI of Supplementary material“**

4. Trials that are “significant” are combined - “Subgroup analyses suggest that only significant interventions had an effect on reducing variability” - but interventions that increase the mean should be separated from those that decrease the mean. The later conclusion that “The variability seems to decrease for treatments that perform significantly better than the reference” suggests a different distinction (better/worse is not the same as larger/smaller because outcomes may be positive or negative) and is not supported by the results presented.

**Thanks for this great contribution. Following your suggestion, we have sought in each primary endpoint for whether improvements in the response correspond to higher (e.g., mobility) or lower (e.g., pain) values. This new factor has been included in the subgroup analysis (see new figures S5-S7 clicking** **here** **or in the Supplementary Material), thus providing an argument for the existence of a "floor" effect in those studies where a lower value corresponds to a better condition. We have added an interpretation of this finding in the Discussion:**

**“This reduced variability could also be due to methodological reasons. One is that some measurements may have a “ceiling” or “floor” effect (e.g., in the extreme case, if a treatment heals someone, no further improvement is possible). In fact, according to the subgroup analysis of the studies with outcomes that indicate the degree of disease (high values imply greater severity; e.g., pain), a greater variance (25%) is obtained in the experimental arm (see Figure S5). However, in the studies with outcomes that measure the degree of healthiness (high values imply better condition; e.g., mobility), the average variances match between arms and do not suggest a ceiling effect.”**

**In addition, we have included this new factor (direction of the improvement) in the** **Shiny app** **.**

**On the other hand, all the significant studies were in favor of the experimental group; therefore, in our context, "statistically significant" is equivalent to "better response in the experimental group". We have specified this statement in the manuscript and we have kept the sentence: "the authors found statistically significant differences between the arms (all of them in favor of the experimental group) in 83 (39.9%) studies"**

5. Abstract, Results: “The adjusted point estimate of the mean ratio (treated to control group) of the outcome variances” is not clear without reading the whole text. Again, defining a term (“outcome variance ratio”?) will help.

**Thanks. Corrected both in the Abstract and the main text:**

**Before [Abstract]: We assessed homoscedasticity by comparing the outcome variability between treated and control arms**

**After [Abstract]: We assessed homoscedasticity by comparing the variance of the primary endpoint between arms through the outcome variance ratio (treated to control group).**

**Before [Abstract]: The adjusted point estimate of the mean ratio (treated to control group)**

**After [Abstract]: The adjusted point estimate of the mean outcome variance ratio (treated to control group) …**

**Before [Methods]: … we fitted a random-mixed effects model using the logarithm of the variance ratio at the end of the trial…**

**After [Methods]: … we fitted a random-effects model using the logarithm of the outcome variance ratio at the end of the trial …**

6. *Table 1, “variability is… increased”: from the text, this means “significantly increased”, which should be clarified.

**Thanks. We have corrected it:**

**Before [Table 1]: increased/decreased**

**After [Table 1]: significantly increased / significantly decreased**

**Interpretation**

The results may be interpreted in many ways, which are sensibly discussed by the authors.

Most importantly, treatment effect homogeneity implies homoscedasticity, but the converse (“homoscedasticity implies treatment effect homogeneity”) is not true: this is demonstrated very nicely in Figure 4. Homoscedasticity is scale-dependent: for example, it may be removed (or created) by a log transformation (mentioned in the Discussion).

1. *The authors omit one alternative explanation of homoscedasticity over time: clinical trial populations have eligibility criteria at baseline which may limit baseline variance. For example, a hypertension trial might recruit patients with baseline SBP between 140 and 159 mm Hg. In this case, variance is very likely to naturally increase over time.

**Thanks again. We have dealt with this in the Discussion:**

**“…it has been observed that the variability in the experimental arm also decreases from baseline to the end of the study, *although this comparison is not protected by randomization; for example, the existence of eligibility criteria at baseline may have limited the initial variance (a hypertension trial might recruit patients with baseline SBP between 140 and 159 mm Hg), leading to the variance naturally increasing over time*”**

2. **The authors’ conclusions ignore the alternative interpretations noted above. Here are some examples which are illogical:

- Abstract, Conclusions: “the variance was more often smaller in the intervention group, suggesting, if anything, a reduced role for precision medicine”, and Discussion: “variability tends to be reduced on average after treatment, thus making precision medicine dispensable in most cases”. This is actually false. If a study finds smaller variance in the treated group then we DO have evidence of treatment effect heterogeneity, and indeed the treatment may be doing exactly what medicine should do - making the sickest better while not harming the less sick.

**Thanks. We agree. We have addressed this point in the general response above. We provide here further specific comments.**

**There is heteroscedasticity of effect leading to reduced outcome variability, such as the one shown in examples E and F of Figure 1. Those cases with reduced variability show situations in which the outcome is “under additional control” at the end. The only mathematical model that we can imagine here is the one with an effect correlated with baseline values: higher effects for higher (worse) baseline values. We can imagine this situation for the “ideal” training program: worse participants at the beginning, which further increases or reduces variability. So, although we agree that this is a theoretical heterogeneity, we do not think that it has any practical implication for “individualizing” the treatment: all patients benefit (although to a different degree) from the intervention; and at the end, all patients are “under additional control”.**

**We have performed some changes in the manuscript in order to clarify this point:**

**Before [Abstract]: the variance was more often smaller in the intervention group, suggesting, if anything, a reduced role for precision medicine**

**After [Abstract]: We found that the outcome variance was more often smaller in the intervention group, suggesting that treated patients may end up pertaining more often to reference or “normality” values and thus would not require further precision medicine. However, this result may also be compatible with a reduced effect in some patients, which would require studying whether the effect merits enduring the side effects as well as the economic costs.**

**Before [Discussion]: variability tends to be reduced on average after treatment, thus making precision medicine dispensable in most cases**

**After [Discussion]: We found that variability seems to decrease for treatments that perform significantly better than the reference; otherwise, it remains similar. Therefore, the treatment seems to be doing what medicine should do –having larger effects in the most ill patients. Two considerations may be highlighted here: (1) as the outcome range becomes reduced, we may interpret that, following the intervention, this population is under additional control; but also, (2) as subjects are responding differently to treatment, this opens the way for not treating some (e.g. those subjects who are not very ill, and so have no scope to respond very much), with obvious savings in side effects and costs**

- Introduction: “If this homoscedasticity holds, there is no need to repeat the clinical trial once a new possible effect modifier becomes measurable” - again, this wrongly assumes the converse stated above.

**In this case, we have softened the sentence by changing the term "need" to "evidence".**

**Before [Introduction]: If this homoscedasticity holds, there is no need to repeat the clinical trial once a new possible effect modifier becomes measurable**

**After [Introduction]: If this homoscedasticity holds, there is no evidence that the clinical trial should be repeated once a new possible effect modifier becomes measurable**

- Discussion: “When both arms have equal variances, then an obvious default explanation is that the treatment is equally effective for all, thus rendering the search for predictors of differential response futile”: this is illogical.

**We are not sure that we understood why this is illogical. Anyway, we have softened the sentence by changing "an obvious default explanation" to "the simplest explanation".**

**Before [Discussion]: When both arms have equal variances, then an obvious default explanation is that the treatment is equally effective for all, thus rendering the search for predictors of differential response futile**

**After [Discussion]: When both arms have equal variances, then the simplest explanation is that the treatment is equally effective for all, thus rendering the search for predictors of differential response futile.**

- Discussion: “For most trials, subjects vary little in their response to treatment, which suggests that precision medicine’s scope may be less than what is commonly assumed”: this is also illogical.

**Again, we are not sure that we understood why this is illogical. Nevertheless, we have referred to the limitations derived from Figure 4.**

**Before [Discussion]: For most trials, subjects vary little in their response to treatment, which suggests that precision medicine’s scope may be less than what is commonly assumed**

**After [Discussion]: For most trials, variability of the response to treatment changes scarcely or even decreases, which suggests that precision medicine’s scope may be less than what is commonly assumed – while always taking into account the limitation previously explained in Figure 4.**

3. *In the light of the above arguments, I find the statement (Abstract, Conclusions) that “Homoscedasticity is a useful tool for assessing whether or not the premise of constant effect is reasonable” to be highly debatable. Logic suggests it gives a lower bound on the extent of usefulness of precision medicine, and the results of this study do not add any more to this.

**We have reduced the ostentatious nature of this phrase, warning the reader that there are limitations to this methodology:**

**“We have shown that the comparison of variances is a useful but not definitive tool to asses if the design assumption of a constant effect holds.”**

4. *The objectives in the Discussion should be the same as those stated in the Introduction.

**Thanks. We have simplified the objectives in the introduction:**

**Before: Our objectives were, first, to compare the variability of the main outcome between different arms in clinical trials published in medical journals and, second, to provide a first, rough estimate of the proportion of studies that could potentially benefit from precision medicine. As sensitivity analysis, we explore the changes in the experimental arm’s variability over time (from baseline to the end of the study). We also fit a random- effects model to the outcome variance ratio in order to isolate studies with a variance ratio outside their expected random variability values (heterogeneity).**

**After: Our objectives were, first, to compare the variability of the main outcome between different arms in clinical trials published in medical journals using a random-effects model; and, second, to provide a rough estimate of the proportion of studies that could potentially benefit from precision medicine. Finally, we explore the changes in the experimental arm’s variability over time (from baseline to the end of the study).**

**Also, we have reordered the whole Discussion section according to these objectives:**

**1) Variability comparison between arms and explanation**

**2) Rough estimate of the studies that potentially benefit from precision medicine (greater variability in experimental arms)**

**3) Variability comparison between arms and explanation provided in your first suggestion of this section.**

**Source data**

1. I had trouble opening the source data both in Excel (since the csv file is in fact semi-colon-delimited) and in Stata (which was thrown by line 80). Could it be provided in a more convenient format or with some notes?

**We have changed the format (now, columns are comma-delimited) both in the** **Shiny app** **and in the** **Figshare** **repository. We also solved the problem with line 80, which included some unnecessary quotation marks (“) in the *Title* field.**

# Response

**Published:** 2018-11-06

**Authors:** Jordi Cortés

**Affiliations:** Universitat Politècnica de Catalunya, Spain

Following your suggestions together with those of the other reviewers, we have updated the manuscript with a new version that aims to emphasize the fact that researchers’ assumption of a constant effect is not clear as long as they do not mention it explicitly. Admittedly, in those studies whose sample size calculation considers some variability in the treatment effect, there is no doubt that this premise has not been considered.
